# Testing a policy intervention in the lab: differences between students and non-students in switching bank accounts

**DOI:** 10.1016/j.socec.2024.102220

**Published:** 2024-08

**Authors:** Antonios Proestakis, Ginevra Marandola, Joana S. Lourenço, René van Bavel

**Affiliations:** a Joint Research Centre, European Commission, Rue du Champ de Mars 21, 1050 Brussels, Belgium.; b Joint Research Centre, European Commission, Edificio Expo, Calle Inca Garcilaso 3, 41092, Seville, Spain

**Keywords:** Economic experiment, Student sample, Switching, Financial behaviour, External validity, Policy intervention

## Abstract

The reliance on student samples has long been a subject of debate in experimental approaches to studying behaviour. We contribute to this discussion by looking at differences in financial behaviour between a student and a non-student sample in three sets of lab experiments conducted in Spain, Germany and Poland (n=857). Participants from both samples switched more often and made better financial decisions after they received a message encouraging them to switch financial service providers. While the size of the effect on switching frequency was comparable between the two samples, the effect on switching quality was significantly stronger on non-students. Further analysis suggests this is due to a better performance of students before the prompt leaving less room for improvement by the reminder. Results suggest that experimental evidence derived from students should be generalized with caution.

## Highlights

• A lab experiment testing the effectiveness of reminders in prompting switching of financial service providers was conducted with students and non-students in three EU countries.
• While the effect of the reminder was significant in both student and non-student samples, its influence was significantly stronger among non-students.
• The effect of the reminder on non-students’ performance was heavily driven by a subgroup – those who never switched – who were overrepresented among non-students.
• Using student samples to produce policy-relevant results can lead to relevant effects going undetected when subgroups – those exhibiting “poorest” behaviour – are of interest.

## 1 Introduction

Behavioural experiments can offer valuable information on potential responses to proposed policy interventions (Camerer, 2015: Falk and Heckman, 2009). Laboratory experiments, in particular, offer the possibility to test different variations of a programme prior to full-scale implementation (Rosch et al., 2021). Yet a common concern is whether these results can be generalized to real world environments (Guala and Mittone, 2005; Levitt and List, 2007a; 2007b; 2008; Schram, 2005).

Laboratory experiments have often relied on university student samples, which are convenient –cheap, accessible, and homogenous. However, students show a greater capacity for abstract reasoning (Belot, Duch, & Miller, 2015) and more rational thinking (Belot, Duch, & Miller, 2015; Sears, 1986), which may lead to a different behaviour from the general population. In this paper we address this issue.

Several experiments comparing students with non-students have shown little or no significant difference in social preferences (Bekkers, 2007; Exadaktylos et al., 2013); willingness to pay for a good (Depositario et al. 2009); and signalling behaviour (Potters & van Winden, 1996). Paolacci et al. (2010) found that MTurk participants behave similarly to university students on a set of non-incentivised experiments drawn from the heuristics and biases literature. Andersen et al. (2010) found no difference in risk or time preferences between students participating in a lab experiment and non-students participating in a lab-in-the field experiment.

However, other experimental studies do show a difference. For example, Anderson et al. (2013), Belot et al. (2015), Cappelen et al. (2015) and Falk et al. (2013) found that students exhibit more self-interested behaviour than the general population, at least in economic experiments. Others find more moderate differences. Masclet et al. (2009) found that students are more risk averse than self-employed people, but not salaried people. Druckman and McDermott (2008) found only weak evidence that students were more risk averse than non-students. A large-scale incentivised survey (Snowberg & Yariv, 2021), reported that university students seem to provide upper bounds on “normative rationality” (they are less generous, more risk neutral, etc.) and on “cognitive sophistication” (they exhibit greater cognitive skills and strategic thinking).

This exploratory study compares students’ and non-students’ choices and responses to an intervention prompting consumers to switch financial service providers. We conducted an experiment (n=857 across three laboratories in Germany, Poland and Spain) that tested the effectiveness of *informative reminders* in reducing inertia – the reluctance to switch to alternative service providers – and improving the quality of switching choices.

The focus of this paper is to evaluate students’ and non-students’ responses to a very specific and realistic policy intervention which may be affected by different observable and unobservable characteristics of the participants. While we measure and control for different observable characteristics such as risk aversion, impatience, financial literacy, sociodemographic variables, etc., we use a control treatment to account for all unobservable characteristics and country effects and study the net effect of the intervention in the two samples. Differently from other papers in the literature, which focus on social, time and risk preferences or abstract reasoning tasks, we use a novel approach which rests in relying on a practical task which simulates a realistic financial decision that participants may have already encountered in their daily life. This allows us to provide evidence based on an exercise that is less abstract and more concrete and therefore the differences we find are less related to students’ higher familiarity with theoretical and abstract tests.

Inertia is a significant phenomenon in retail finance (Adams et al., 2015; Adams et al., 2016; Adams & Smart, 2017; Wruuck, 2013), and merits attention because, beyond consumers’ missed opportunities, it can lead to a reduction in competition (Farrell & Klemperer, 2007; Klemperer, 1995; Waterson, 2003). On the other hand, increasing switching *per se* is not enough as a policy objective if the change fails to make consumers better off.

In our experiment, the reminder also contained some additional information on the pros and cons of switching, hence an *informative* reminder. In financial services, inattention is often singled out as the culprit for inertia (Andersen et al., 2015; Bajo & Barbi, 2015; Branzoli, 2016; Dambe, 2013). In other words, consumers fail to consider information about the market or about products that might increase their welfare, or fail to notice an opportunity to act, like an annual switching opportunity (Grubb, 2015; Heiss et al., 2021). Sometimes, events such as a rise in interest rates will spark their attention and make them look for better offers (Lukas & Nöth, 2019). Other times, reminders such as text messages, emails and letters will encourage consumers to make active decisions on loans and switching bank accounts (Adams et al., 2015; Adams et al., 2016; Adams & Smart, 2017; Barr, Bird, & Castleman, 2021). In the present study, a standardised informative reminder is used as an instrument for testing whether policy interventions of this type have a differential effect in the two samples. This study aims to inform the scientific community and policymakers about whether laboratory experiments using convenience student samples can serve as a foundation for developing policy interventions before full-scale implementation.

We found that the *informative* reminder was effective in improving switching choices both quantitatively (switching more often) and qualitatively (switching to optimal choices) among both non-students and students. In contrast to Snowberg & Yariv (2021), who only reported population-level shifts, we identified a subgroup of participants – those who never switched – who were overrepresented among non-students compared to students. The effect of the reminder on non-students’ performance was heavily driven by this subgroup of participants.

The study contributes to the debate on the use of student samples for assessing policy interventions by focusing not only on behavioural differences (i.e. preferences, strategic decisions) between a student and a non-student sample, but also on differences in the effectiveness of the treatment in these samples. Our study relates closely to a recently published study by Barr et al. (2023) on the sensitivity of laboratory findings to changes in experimental samples (and settings). By means of a distributive justice experiment (Barr et al., 2016) the paper evaluates the treatment effect on two different populations (students vs. non-students) and two settings (experimental survey and lab experiment). The authors conclude that within each population the effect is very similar in the two settings. Although not the focus of this study, a similar treatment effect has been reported in the two samples (in the lab setting). Along the same lines, Bader et al. 2021 found that data obtained from standard participant pools differ significantly from those from the broader population, and the direction of these behavioural differences between games is remarkably robust to changes in samples and settings.

The rest of the paper has the following structure: Section 2 presents details of the experimental design and protocol. Section 3 shows the results of the experiment. Section 4 discusses these results and concludes.

## 2 Sample and experimental design

### 2.1 Sample

Our sample had 357 university students and 500 non-students. The laboratory experiment was carried out in Spain (Jaume I University in Castellón), Germany (Ludwig Maximilian University of Munich), and Poland (University of Warsaw). Students were drawn from the three university subject pools, and non-student participants were recruited either by external recruitment companies (Germany and Spain) or by a university (Poland). Non-students were recruited to be as close as possible to the respective country's population in terms of gender, age and educational level (further details in Appendix A1). These three countries were chosen to capture (and control for) the geographical and cultural variety of Europe (i.e. Central European, Mediterranean and Eastern European)^1^ with the final aim to provide policymakers with broader and more representative evidence on European citizens' behaviour.

### 2.2 Experimental design

The aim of the experiment was to compare financial behaviour between the sample of students and non-students, in particular switching financial service providers in the presence of an informative reminder. To this end, the experiment included a distractor task, a switching task, and mock bank account contracts. Participants were given a limited time to complete the distractor task and, during this period, they also had the opportunity to check available bank accounts offers and switch. Switching to a better contract (than the one allocated at the start) meant higher earnings. The experimental design included variable monetary incentives linked with performance, which were expressed in points, paid into the participant's mock bank account, and converted in local currency at the end.

### 2.3 The counting/distractor task

**Fig. 1:**
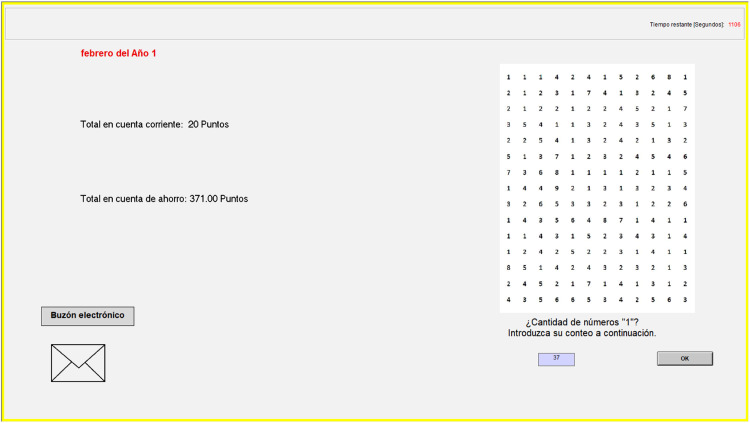
Print screen showcasing the design of a period or “month”.

Participants had to repeatedly perform a distractor task, which required them to invest a minimum effort to earn a monetary compensation. Specifically, participants moved through different “months” in the experiment. In each “month”, a matrix filled with random one-digit numbers (1-9) was displayed on the screen and they had to count all the "1s" in the matrix (Fig. 1). This counting task is a well-established real-effort task (Carpenter, Huet-Vaughn, 2019), and was selected as it does not require any prior knowledge, there is little learning possibility (Abeler, Falk, Goette, & Huffman, 2011), and performance is mainly a function of effort (Brooks, Stremitzer, & Tontrup, 2017).

In this study, if participants gave a wrong answer in the counting task, they were asked to input a new answer until it was correct (a max of 100 attempts were allowed within the same month). Once the counting was correct, they moved to the screen with the next matrix and earned points for the correct answer. Participants could only move forward and earn points for solving the matrix if they had provided the correct answer. They could also move forward by making 100 incorrect attempts, albeit without earning any points for that matrix. This task and its associated reward were designed to simulate a “monthly” salary.

Participants were assigned a mock bank contract at the beginning of the experiment, which included two accounts: a payment (current) account and a savings account (see Appendix B for details). In each month, participants’ earnings from the counting task (i.e. their “monthly salary”) were paid into their payment account. Additionally, they had been endowed with points in their savings account at the start of the experiment, which earned interest at the end of each “month”. The number of points that participants had in their payment account and in the associated savings account were visible on the screen in each month (top left of Fig. 1). It was not possible to make transfers between the two accounts. Participants had 25 minutes to solve up to 40 matrices, representing 40 “months” (January Year 1 to April Year 4).

### 2.4 The switching task

Although participants were initially assigned to one bank account contract, they had the possibility to browse and switch to another contract throughout the experiment. Participants could switch as many times as they wished, but each time they switched they incurred a small cost (see Appendix B for details). To observe switching behaviour, the experiment offered participants several alternative account contracts that they could switch to.

**Fig. 2:**
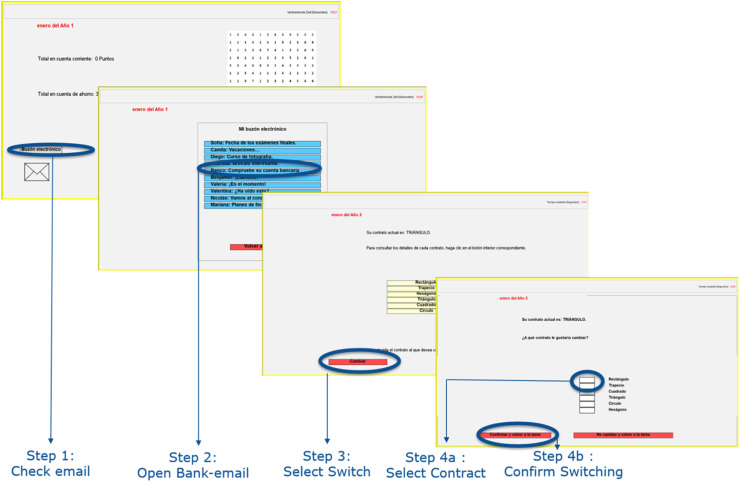
Illustration of the switching process.

Fig. 2 illustrates the steps required for switching, from exiting the main “monthly” screen and entering the mailbox (step 1) to selecting the contract and confirming switching (step 4). Information on each individual contract could be gathered in step 3, by clicking on each of the yellow contract buttons that were visible on the screen. The switching process required several steps with the goal to make it time consuming for participants and to simulate the administrative steps they would take in real life. Participants had to take these additional switching actions while performing the counting task and therefore their overall performance might be also affected by their ability to multitask (Buser & Peter, 2012).

Switching could lead to additional payoffs if participants switched at the right time to a contract with a lower monthly fee and/or a higher interest rate. Specifically, while the contract assigned by default to participants at the start was the best option at the beginning, it eventually (after 10 months) stopped being the best choice. That is, the assigned contract in the experimental environment was equivalent to a teaser contract and participants’ optimal response would be to switch to another one once the teaser period elapsed. Then, later in the experiment, a new, more profitable, contract became available in the market and the optimal choice would be to switch a second time. The experiment recorded whether participants switched at the right time to the right contract, for each of the two switching instances.

Monetary gains from switching were high enough so that switching efficiently would lead to substantially higher earnings (Table 1). Namely, participants who selected the best contract at each period in time and solved all the matrices would earn 1.6 times the earnings of those who never switched and did not solve all the matrices. Moreover, on average those who switched at least once earned 1.3 times the earnings of those who did not switch.

**Table 1:** Earnings from variable payment (in euros) for different scenarios.

	No switching & completion of 40 months	No switching average payoff	Switching optimally & completion of 40 months	Switched at least once average payoff
Germany	12.38	11.81	20.08	15.54
Spain	10.89	10.00	17.67	13.33
Poland	6.19	5.54	10.04	8.5

***Note****:* Columns 1 and 3 show the maximum possible payoffs for participants who completed the total number of matrices. By design, potential payoffs were higher for those who switched optimally (column 3) with respect to those who never switched (column 1). Columns 2 and 4 show the actual payoff in the experiment for those who never switched (column 2) and for those who switched at least once (column 4). Switching was beneficial in each country.

In summary, within the 25 minutes, in addition to the counting task, participants had the opportunity to check available bank accounts offers and switch. Participants were provided with the alternative bank accounts prior to the start of the experiment during the instructions (i.e. they were given 5 minutes to look at all six contracts in paper version before the documents were taken back). Further, upon the start of the experiment, each contract could again be visualised on screen by following the steps described in the instructions (in brief, going to the mailbox, selecting open bank e-mail, and then selecting check information about alternative accounts and clicking on the name of the desired contract) (see Appendix B.2 for further details). Each contract contained information about (a) the name of the contract (triangle, square, etc.), (b) the monthly current account fee (in points), (c) the monthly interest earned (percentage), (d) the contract availability period (starting month or points requirement) and (e) other distracting information (e.g. payments and cards’ details, which were identical for all accounts). Better offers meant higher earnings, because of lower payment account fees, and/or higher saving account interest rates. In particular, all participants were assigned the “Triangle” contract (teaser contract, with the highest interest rate until Month 10) by default at the start of the experiment. Once the teaser period ended, “Square” was the optimal choice. After that, once their current account balance was at least 388 points (i.e. by Month 20 if their behaviour had been optimal), “Trapezium” became the optimal choice (see Appendix B.1 for an overview). The current account balance was always available to participants as it was shown on the main task screen (Fig. 1).

On the other hand, switching and shopping around diverted time from the counting task, so that switching had an opportunity cost on top of a small switching fee. Indeed, the number of available matrices was set higher than the number of matrices that an average participant was expected to solve^2^. This enabled us to examine the extent to which people would optimize or not their performance since the time limit imposed a trade-off, that is, engaging in switching could be profitable but involved foregoing time/benefits from the counting task. Similarly, in real life contexts people repeatedly face trade-offs, such as, for example, choosing between engaging in one activity or another, since time is a scarce resource (Haghpour et al., 2022).

At the end of the experiment, the accumulated points from the savings and payment accounts were exchanged for real money and given privately and anonymously to participants. Across different labs exchange rates were adjusted according to each country's purchasing power parity. The exchange rate was the same for both students and non-students^3^.

### 2.5 Experimental conditions

Participants were randomly allocated to one of two experimental conditions: control or treatment.^4^ The difference was in the bank statement that participants received every 12 “months”. Specifically, after solving 12 and 24 matrices, and before moving to the next one, participants saw a different screen. The screen included a box at the top that was either blank (control) or contained an informative reminder message that encouraged them to switch (treatment). The reminder comprised four sentences, together composing a narrative in favour of switching. Translated to English, they read: “Everybody should switch because there are contracts that after some time are not as favourable as at the beginning and others that the more money you have the cheaper they are.”; “Without switching one can solve even more tasks.”; “But it is worth switching to get the lowest fee, and a good %.”; “Switching costs are not too high. By switching you can gain reasonable amounts in the following months”.

In both control and treatment groups, participants could not close the annual statement and continue completing the tasks until at least 10 seconds from the appearance of the statement on the screen had elapsed. The aim was to ensure that participants in the treatment group were exposed to the reminder.

### 2.6 Socio-demographic variables and behavioural preferences

At the end of the experiment, participants filled out a questionnaire to collect socio-demographic as well as measures of risk attitude, time preferences and financial literacy^5^. Risk attitude was measured through an incentivised task, in which participants had to decide how much they would like to invest in a risky project. The amount invested was a measure of participants’ risk aversion (Charness & Gneezy, 2010). Time preferences were measured through a non-incentivised set of questions asking participants to reveal their preference between a lower amount soon and a higher amount later. We took this time preference to be a measure of impatience, and this is the term we use throughout the paper (Andersen et al., 2008). Financial literacy was measured by the response to three multiple-choice questions (Lusardi & Mitchell, 2008). An overview of the full experimental protocol and the English version of the instructions can be found in Appendix B2.

## 3 Results

### 3.1 Study sample

Table 2 presents summary statistics of the two samples. Students were (naturally) younger and more homogenous in age than non-students. Also, by construction, there were differences in education level between students and non-students, as most of the university students had only attained secondary school diploma by the time of the experiment. There were no differences in gender and financial literacy between the two groups; likewise, time preferences and risk aversion were also not significantly different (as in Harrisson et al., 2002 and Andersen et al., 2010). However, students required fewer attempts to correctly answer three multiple-choice questions that checked participants’ understanding of the experimental procedure and payoff system. This may reflect greater ability to cognitively process and retain relevant task information, as well as students’ greater experience with laboratory experiments and associated instructions and procedures.

**Table 2:** Main characteristics of the student and non-students’ samples.

	Students (n = 357)		Non-students (n = 500)		Average Difference
	Mean	Std. dev.	Mean	Std. dev.	*p-*Value
Age (years)	21.28	2.15	41.47	10.81	<0.001
Male	0.50	0.50	0.50	0.50	=0.995
Education					
None/primary education	0	0	0.06	0.23	<0.001
Secondary education	0.62	0.49	0.47	0.50	<0.001
Undergraduate	0.24	0.43	0.21	0.40	=0.224
Master/Ph.D.	0.14	0.35	0.27	0.44	<0.001
Financial literacy (0‒3)	2.34	0.87	2.37	0.80	=0.514
Impatience (0‒1)	0.10	0.31	0.11	0.31	=0.767
Risk aversion (0‒1)	0.51	0.50	0.51	0.50	=0.949
Wrong Answers (0‒4)	2.21	1.44	2.73	1.53	<0.001

***Note**: Age* is the age of participant in years. *Male* is the proportion of men in the sample. *None/primary education* is the proportion of participants with no or primary education. *Secondary education, Undergraduate* and *Master/PhD* are the proportions of participants with Secondary, Undergraduate and Master/PhD respectively (see Appendix A3 for details). *Fin. Literacy* goes from 0 to 3 that is the number of correct answers to financial literacy questions; *Very impatient* are those who never change the present for a future higher compensation, dummy equal to 1; *Risk Aversion*: all those people who have investment<=40, dummy equal 1 for risk averse participants. *Wrong Answers,* the variable is 0 if the participant gets all the answers at the first trial, 1 if 1 additional attempt is needed, 2 for two attempts, 3 for 3 attempts, 4 if at the third attempt they were still wrong and therefore they need to answer again after having received the correct answers.

### 3.2 Differences in performance between students and non-students in the control condition

The key outcome measures capturing participants’ switching behaviour were *switching frequency* (how many times participants switched account contract), and *optimal choice* (the percentage of participants who held the best available contract in each period). *Inertia* (the percentage of participants who never switched contract) was further explored as a subcase of switching frequency.

**Fig. 3:**
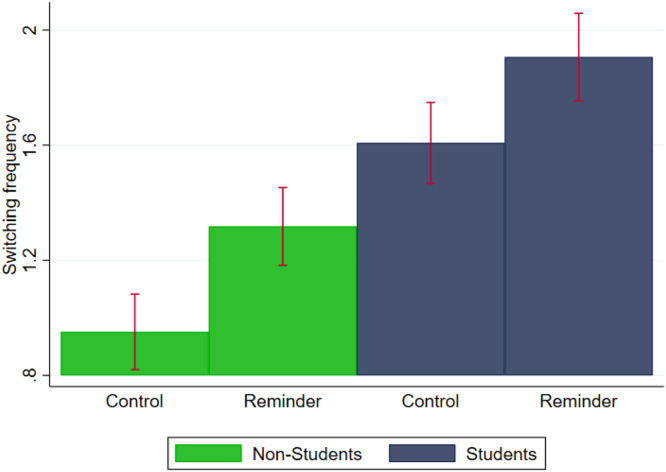
Switching frequency for non-students and students.

In the control condition, students were faster than non-students in the distractor task, such that they were able to solve more matrices (Mann-Whitney test*,* p < 0.001) and thus reach a greater number of “Months”. Specifically, they reached, on average, Month 32 (SD = 6.46) and Month 28 (SD = 6.98), respectively. These results are consistent with non-student's lower information processing speed (e.g. Deary & Der, 2005; Hertzog & Bleckley, 2001) as well as lower familiarity with the digital environment. With regards to switching behaviour, students switched significantly (Mann-Whitney test, p<0.01) more often than non-students: 1.61 times (SD = 0.94) vs. 0.95 times (SD = 1.04) respectively (Fig. 3; see also Table 3, column 1 for additional analysis).

**Table 3:** The effect of reminder on switching frequency-Poisson Regressions.

	(1)	(2)
	Switch (Full)	Switch (Full)
Student	.354***	.397***
	(.051)	(.08)
Reminder	.234***	.273***
	(.051)	(.081)
Rem_Std		-.074
		(.102)
Male	.147***	.144***
	(.052)	(.052)
Fin. Literacy	.181***	.182***
	(.036)	(.036)
V. Impatient	-.151	-.15
	(.094)	(.095)
Risk averse	.014	.014
	(.05)	(.05)
Wrong answers	-.073***	-.072***
	(.016)	(.016)
# Months	.014***	.014***
	(.004)	(.004)
Constant	-.677***	-.703***
	(.172)	(.176)
Observations	857	857
Pseudo R^2^	.059	.059
Country Dummies	Yes	Yes

***Note:*** Poisson regressions with robust standard error (in parentheses). The dependent variable is the total number of switches or switching frequency. *Reminder* is a dummy (= 1) if the observation comes from the reminder treatment and student is dummy (=1) if the observation comes from students’ sample. *Male* is =1 if participant is male; *Fin. Literacy* goes from 0 to 3, that is, the number of correct answers to financial literacy questions; *Age* is the age of participant in years. *Very impatient* are those who never changed the present for a future higher compensation, dummy equal to 1. *Risk Aversion*: all those people who have investment<=40, dummy equal 1 for risk averse participants. *Wrong Answers,* the variable is 0 if the participant gets all the answers at the first trial, 1 if 1 additional attempt is needed, 2 for two attempts, 3 for 3 attempts, 4 if at the third attempt they were still wrong and therefore they need to answer again after having received the correct answers. *#Months* is the number of months (matrices) reached by the participant. In model (2) Rem_Stud is the interaction between Reminder and Student. We controlled for country in all models. *** p<.01, ** p<.05, * p<.1

**Fig. 4:**
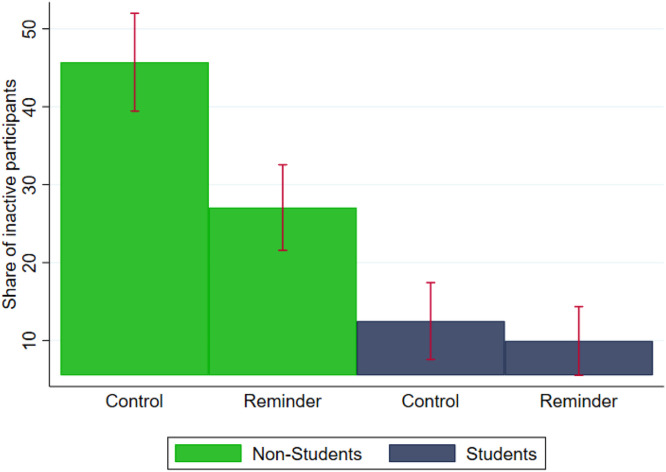
Inertia for students and non-students.

There were also striking and highly significant differences in the share of participants displaying *inertia* in the control condition (proportion test, p <0.001): only 12.5 % (SD = 0.33) of students never switched, compared to 45.7 % (SD = 0.50) of non-students (Fig. 4).

With regard to the quality of switching choices, students behaved more optimally (Mann-Whitney test, p <0.01), such that the proportion of months in which participants held the best available contract was higher for students (mean 0.64, SD = 0.24) than for non-students (mean = 0.57, SD = 0.22; see also Table 4, column 1).

**Table 4:** Effect of the reminder on optimal choice- Multilevel Logit Regressions.

	(1)	(2)
	*Optij*	Optij
Reminder	-.345***	-.527***
	(.124)	(.163)
afterAS1	-.947	-1.504
	(1.109)	(1.104)
Student	.434***	-.471**
	(.119)	(.186)
ReminderXAS	.585***	.787***
	(.078)	(.107)
rem_std		.434*
		(.252)
AS_std		1.247***
		(.114)
rem_AS_std		-.439***
		(.156)
Male	.291**	.291**
	(.115)	(.116)
Fin. Literacy	.235***	.239***
	(.073)	(.073)
V. Impatient	-.162	-.158
	(.184)	(.186)
Risk averse	-.188*	-.183
	(.114)	(.115)
Wrong answers	-.145***	-.149***
	(.039)	(.039)
# Months	.013	.015*
	(.009)	(.009)
Germany	.033	.029
	(.139)	(.14)
Poland	.317**	.3**
	(.143)	(.144)
Constant	-.190	.110
	(.970)	(.969)
Observations	25293	25293
Chi^2^	147.142	314.374

***Note***: Multilevel (mixed effects) two-way crossed-effects (subjects(i) X periods(j)) logit regression models on the binary dependent variable “Optimal Contract”, equal to one if the participant holds the best available contract in the current month, 0 otherwise. Reminder is a dummy variable capturing the treatment effect; AfterAS1 is a dummy indicating the months after the first annual statement (i.e. month >12); ReminderXAS is the interaction of the two dummies. We control for Male, Fin. Literacy, Very impatient, Risk Aversion, Wrong Answers, Country and #Months as defined in Table 3. In Columns 2 we include the variable rem_AS_std capturing the triple interaction of Reminder, afterAS1 and Student and the variables rem_std and AS_std to capture the interactions of variables Reminder and afterAS1 with the variable student, respectively. The panel dataset is unbalanced since not all participants managed to solve all matrices and therefore go through all 40 months. Since we have a panel, the total number of observations is the product of the number of subjects (N) and the corresponding number of months for each subject.

Taken together, these results suggest that students in the control condition performed significantly better than non-students in both the distractor and the switching tasks. This is in line with Snowberg and Yariv (2021), who find that the student population is closer to the ideal of normative rationality and exhibits greater cognitive sophistication than the general population. It also aligns with Brocas et al. (2019) who, by comparing the choices of students and older adults, find that older adults display less consistent decision-making than students in complex situations, due to age-related deterioration of neural faculties responsible for working memory and fluid intelligence.

### 3.3 Effect of the reminder on switching frequency

The informative reminder had a significant (p < 0.01) effect (see Table 3) and increased the total number of switches by 26 % across the full sample (odds ratio of the Reminder term was 1.26). Switching frequency was moderated by participants’ characteristics, beyond being a student or not. Men (vs. women), the more (vs. less) financially literate participants and those who solved more (vs. fewer) matrices/months^6^ switched significantly more often (p < 0.01) (Table 3, column 1).

Looking at the different samples, the informative reminder significantly increased switching frequency among students (Mann-Whitney test, p = 0.039) and non-students (Mann-Whitney test, p < 0.001). Switching frequency increased from an average of once in the control condition to 1.4 times in the treatment condition among non-students, and from 1.6 times in the control condition to 1.9 times in the treatment condition among students (Fig. 3). The informative reminder had a comparable effect on students and non-students (Table 3, column 2), as shown by the non-statistically significant coefficient on the interaction term (*Rem_Std*) between *Reminder* and *Student*.

### 3.4 Effect of the reminder on optimal choice

**Fig. 5:**
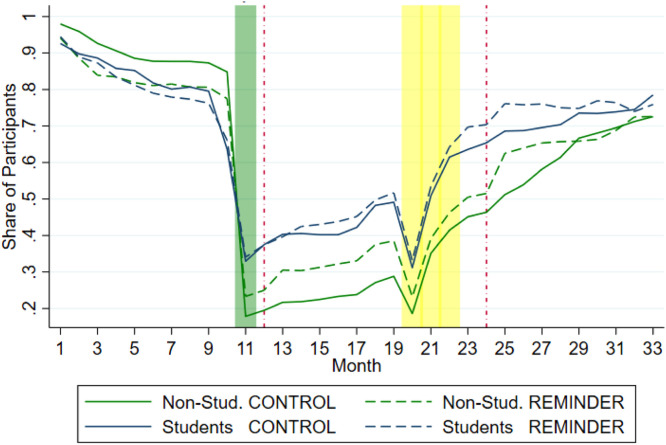
Percentage of participants holding the optimal contract in each month. ***Note**:* The green and yellow shaded areas represent the optimal switching points (month 11 and months 20-22) (see Table B1 in appendix). Red dotted lines (months 12 and 24) indicate the timing of the switching reminder presented to non-students and student participants in the treatment/reminder condition. The green lines represent the share of non-students participants holding the best contract in each month, and the blue ones the share of students. For both non-students and students, the continuous lines represent the control condition, and the dotted lines represent the reminder or treatment condition.

While switching frequency is an important indicator, and a proxy of consumers’ ability to exercise their choice, it does not provide evidence on the quality of participants’ switching choices. To gain further insights into this, we mapped out the theoretically optimal decision path for consumers and measured to what degree consumers adhered to it. We called this *optimal choice* (i.e. holding the best available contract in a given month). Fig. 5 shows the proportion of participants, for each experimental group, holding the optimal contract in each month.

Fig. 5 shows a sharp decline in *optimal choice* at Month 11. This was the month when the interest rate on the initial teaser contract dropped from 6 % to 2 %, making it less beneficial. It was the first point in time when switching became the optimal choice (before then, the optimal choice was to keep the initial contract). Yet many participants failed to do so. In the control group, the percentage of non-students holding the best available contract dropped from 84.8 % in Month 10 to 17.8 % in Month 11. A similar, but less dramatic, drop was observed among students, from 63.6 % to 33.0 %.

The informative reminder, which appeared for the first time in the annual statement at Month 12 for those in the treatment group, increased *optimal choice* relative to the control for both students and non-students. Fig. 5 shows a distinct pattern across the two samples, such that the effect was higher for non-students than for students, at least until Month 28^7^. This contrasts with the effect of the reminder on *switching frequency*, which showed no differences between the two samples.

The effect of the reminder on *optimal choice* is confirmed in the multilevel (mixed effects) model^8^ shown in Table 4 where each observation is the participant's choice in a given month. In all the models of Table 4, we add, to the explanatory variables of Table 3, the dummy variable *afterAS1* to indicate the ‘treatment period’ which corresponds to all months after Month 12, after participants have seen the first annual statement (with or without informative message). The effect of the treatment (i.e. the effect of the informative message after the annual statement) is captured by the variable *ReminderXAS* which is the interaction of two dummies; the treatment group (*Reminder*) and the treatment period (*afterAS1*).

Results (column 1) confirm that the effect of the reminder on *optimal choice* is positive and highly significant (p < 0.01) and indicate that the reminder increases the likelihood of making an optimal choice by 79 % relative to no reminder (control group; odds ratio of the interaction term *ReminderXAS* is 1.79).

Regarding socio-demographic variables, the regression shows that males and more financially literate participants are better at identifying the best available contract. Not surprisingly, those who performed worse in the comprehension questions (more wrong answers) also did worse in the switching task. Finally, for the first time, we also found country effects. Participants in Poland performed significantly better than participants in Spain and this was true only in the students’ sample. However, German and Spanish students did not differ in optimal behaviour. Finally, repeating the intervention (second annual statement, presented between Months 24 and 25) did not have a statistically significant effect in increasing further the percentage of participants who switched to the right contract (Table C5 in Appendix C).

To analyse if the reminder had a differential effect in the two populations, we run a model with a triple interaction to account for pre-treatment differences in performance across the two samples (Table 4; column 2). Specifically, despite random allocation of participants to the two conditions and the experimental setting in both being identical for all periods before AS1 (henceforth control-periods), inspection of Fig. 5 suggests that there are differences between conditions in the participants’ optimal behaviour in the control-periods. This is confirmed by the coefficient of *Reminder,* capturing the performance of participants before AS1, which is negative and highly significant as reported in column 1 in Table 4. In other words, participants in the control group make more optimal choices than those in the treatment group in the control-periods notwithstanding the random allocation and the identical situation^9^.

Further, results confirmed that the effect of the reminder on *optimal choice* of non-students is positive and highly significant (*ReminderXAS*, p < 0.01). Importantly, while the positive effect of the reminder is still significant for students (i.e. *ReminderXAS+rem_AS_std* = 0.348, p<0.01), it is smaller (triple interaction coefficient *rem_AS_std*, is negative and significant at p<0.01) (Table 4; column 2). In particular, making an optimal choice is 36 % less likely for students than non-students (odds ratio of the triple interaction term *rem_AS_std* is 0.64) after receiving the informative reminder. Finally, we found no country differences in the effectiveness of the reminder (i.e. the triple interaction among reminder, AS1 and country, was not significant).

### 3.5 Effect of the reminder on inertia

The effect of the informative reminder on *switching frequency* and *optimal choice* for non-students largely depend on its effectiveness for reducing *inertia* (the percentage of participants who never switched contract). The reminder reduced *inertia* among non-students (45.7 % vs 26.7 %), but not among students (Fig. 4).

Table 5 reports the effect of the reminder on the subsample of students and non-students who had not switched before Month 12 (i.e. who displayed inertia before our intervention took place). Regression estimates confirm that the reminder was only effective in reducing inertia among non-students (columns 1 and 2). This is also confirmed by model 4 which includes observations from both samples and the interaction (*rem_std*) between *Student* and *Reminder*. While *Reminder* (capturing the effect on non-students) is highly significant, the linear combination between *Reminder* and *rem_std* (capturing the effect on students) is not significant (p=0.573). Inertia is also significantly smaller for students (as indicated by *Students* regressor) in both models 3 and 4. Note that in these two models, *age* and education dummies are not included to avoid multicollinearity problems with *Students*.

**Table 5:** The effect of reminder on inertia in non-students and students -Logit Regressions.

	(1)	(2)	(3)	(4)
	Non-Stud.	Students	ALL	ALL
Reminder	-.997***	-.217	-.748***	-.934***
	(.258)	(.41)	(.207)	(.239)
Student			-.867***	-1.192***
			(.254)	(.341)
rem_std				.698
				(.485)
Male	-.031	-.268	-.123	-.102
	(.27)	(.428)	(.213)	(.214)
Fin. literacy	-.629***	-.075	-.425***	-.433***
	(.159)	(.25)	(.125)	(.124)
V. Impatient	-.106	.998	.296	.341
	(.401)	(.613)	(.314)	(.317)
Risk averse	.454*	.17	.404*	.378*
	(.269)	(.439)	(.212)	(.214)
Wrong answers	.014	.105	.047	.032
	(.096)	(.153)	(.079)	(.08)
# Months	-.092***	-.053	-.095***	-.097***
	(.023)	(.035)	(.017)	(.017)
Age	.04***	-.029		
	(.013)	(.138)		
_cons	2.639**	1.091	3.664***	3.862***
	(1.047)	(3)	(.685)	(.696)
Observations	339	147	486	486
Pseudo R^2^	.215	.085	.19	.194
Country Dummies	Yes	Yes	Yes	Yes
Education Dummies	Yes	Yes	Yes	Yes

**Note**: Logit regression with robust standard errors (in parentheses). The regressions only include participants that are inactive before the reminder. The dependent variable is =1 if the participant never switched. In column 1, the sample is non-students, in column 2 the sample is student sample and in column 3 and 4 the analysis is for both samples. *Reminder* is a dummy = 1 if the observation comes from the treatment; Student is a dummy=1 if the observation is from the students’ sample and rem_stud is the interaction between Reminder and Student. We control in all regressions for Male, Fin. Literacy, Very impatient, Risk Aversion, Wrong Answers, Country and #Months as defined in Table 3. In regressions 1 and 2 we also control for age and educational level *** p<.01, ** p<.05, * p<.1

Results in columns 1 and 2 show that other observable characteristics explained inertia, but only for non-students. Among non-students, participants who had greater financial literacy and completed more months (or matrices) exhibited less inertia. Moreover, we found a statistically significant positive association between age and inertia. To illustrate, a participant aged 40 exhibits approximately threefold higher odds of becoming inactive compared to a 20-year-old participant, while a 60-year-old participant's odds are approximately nine-fold higher.

Additional analysis (see section C3 of the Annex for details) revealed heterogeneity in the effect of the informative reminder on inertia among non-students. It reduced inertia among females (but not males), and among participants who were slower in the counting task (below the median). The latter result suggests that the effect of the reminder was stronger on those who performed worse in the distractor task, that is, those participants for which the trade-off between switching and solving the matrices was higher.

### 3.6 Differences in performance between active and inactive participants

The heterogeneous effect of the reminder on non-student's inertia invites a comparison of active and inactive participants to understand if they differ in any particular socio-demographic or behavioural measure. We focus on the non-student sample by comparing the behaviour of active and inactive non-students in the control and in the reminder conditions separately. Later, towards the end of this subsection, we briefly comment on students’ behaviour and use pooled data from all samples to highlight overall differences between active and inactive participants.

In the control condition (Table 6, left panel), results show some statistically significant differences, such that, on average, participants in the inactive subsample are older, include a higher proportion of women, have lower financial literacy, and lower cognitive ability (i.e. greater number of wrong answers to comprehension questions and lower number of matrices solved). Moreover, the higher the number of solved matrices by non-students, the lower the inertia.

**Table 6:** Main characteristics of the active and inactive non-student sub-samples.

	Non-students - Control					Non-students - Reminder				
	Active		Inactive			Active		Inactive		
	(n = 133)		(n = 112)			(n = 186)		(n = 69)		
	Mean	Std. dev.	Mean	Std. dev.	*p-*Value	Mean	Std. dev.	Mean	Std. dev.	*p-*Value
Age	38.21	10.15	46.44	10.48	0.000	39.12	10.15	46.04	9.99	0.000
Male (proportion)	0.58	0.50	0.40	0.49	0.006	0.50	0.50	0.50	0.50	0.979
No/primary education	0.04	0.19	0.09	0.29	0.093	0.03	0.18	0.12	0.32	0.009
Secondary education	0.43	0.5	0.54	0.5	0.094	0.42	0.49	0.57	0.5	0.038
Undergraduate	0.23	0.42	0.15	0.36	0.110	0.26	0.44	0.10	0.30	0.007
Master/Ph.D.	0.3	0.46	0.22	0.42	0.171	0.29	0.46	0.22	0.42	0.244
Financial literacy	2.56	0.67	2.07	0.88	0.000	2.56	0.67	2.01	0.96	0.000
Impatience	0.08	0.28	0.15	0.36	0.090	0.09	0.29	0.14	0.35	0.217
Risk aversion	0.50	0.50	0.60	0.49	0.139	0.46	0.50	0.52	0.50	0.399
Wrong Answers	2.74	1.46	3.37	1.09	0.000	2.34	1.67	2.77	1.55	0.076
No. Months	30.41	6.10	24.50	6.32	0.000	28.78	6.70	25.46	7.63	0.000

**Note***:* Age, Male, Fin. Literacy, Age, Very impatient, Risk Aversion, Wrong Answers, and #Months as defined in Table 3. No/primary education, Secondary education, Undergraduate, Master/PhD, as defined in Table 2 and Appendix A3.

In the reminder condition (Table 6, right panel), results show that those who are inactive are still older, have lower financial literacy and solve fewer matrices as compared to active participants in the same condition. In the reminder, differently from the control, inactive participants have a lower level of education, which suggests that the reminder tends to be more effective on participants with higher education (i.e. subjects with undergraduate education compared to subjects with secondary education or lower). By contrast, while the inactive sample included a greater proportion of women in the control condition, there is no such difference in the reminder condition. This is consistent with the heterogeneous effect of the reminder by gender, such that it has a positive effect on women but not men (see Table C3, appendix). The same is true for cognitive ability. While the number of *wrong answers* given by inactive subjects was significantly higher compared to the ones given by active subjects in the control condition, this difference vanished in the reminder condition. The reminder message made subjects with lower performance in the comprehension questions to take a switching action. However, as shown in Table 6 (above) and in column 3 of (Appendix C), active non-students participants in the reminder condition solved significantly fewer matrices than those in the control condition. The effect was probably the result of the reminder inducing those participants who were less familiar with the experimental environment and, thus slower, to give it a try and switch.

Differences between active and inactive student participants were also examined (but not shown here) and revealed that among students only risk aversion played a role. Namely, in the reminder treatment, 83 % of active participants are risk averse, while in the control condition, 47 % of active participants are risk averse. In the reminder treatment only 38 % of inactive participants are risk averse (p =0.003) and 50 % of the inactive participants are risk averse in the control treatment. This finding suggests that the reminder had a stronger effect on risk averse students.

Finally, when we pooled data from all the different samples, encompassing a total of 221 inactive and 636 active participants, our findings revealed a significant (Mann-Whitney test) disparity in performance. Inactive participants exhibited markedly poorer performance in solving the financial questions, matrices, and tasks related to comprehension (p<0.01 in all instances). Furthermore, they demonstrated a significantly higher degree of impatience and risk aversion (p<0.001).

**Fig. 6:**
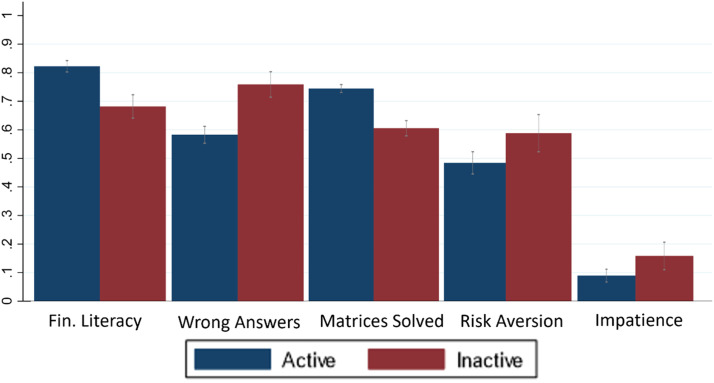
Performance (with 95 % confidence intervals) of Active and Inactive subjects (pooled data) in cognitive tasks and behavioural measures. ***Note****:* The tasks include number of correct answers in Financial Literacy tests, number of wrong answers in Instructions’ Comprehension test and number of matrices solved, all normalized between 0 (min) and 1 (max). Behavioural Games included tasks for eliciting Risk Aversion and Impatience as defined in Fig. 3.

To visually represent these distinctions, Fig. 6 showcases the performance of active participants (depicted by blue bars) and inactive participants (represented by red bars) across various aspects, including financial literacy, instructions comprehension (i.e. wrong answers), and matrices solving, as well as in behavioral measures of risk aversion and impatience. The data has been normalized between 0 (minimum) and 1 (maximum) to facilitate meaningful comparisons.

### 3.7 Differences in performance between students and non-students (active subsample)

Inducing participants to switch does not necessarily make them better off if they fail to make the appropriate choices or if they switch more often than necessary. For some participants, no action may be more beneficial than switching. In order to exclude the existence of these undesired effects in our sample, we compare performance of active participants in the control and in the reminder treatment. Basically, we compare performance between those who switched (by excluding those who never switch) in the control, where the switching choice by default is not manipulated, and those who switched in the reminder who can be either autonomous switchers (those who would have switched regardless of the reminder) or induced switchers (those who switched prompted by the reminder). Given that in the student sample the reminder did not significantly affect the level of inertia, which was very low in the first place, we assume that on average all active students in the reminder treatment are autonomous switchers. On the other hand, given the significant effect of the reminder on inertia in the non-student population, we may expect that active participants in the reminder sample consists of both autonomous and induced switchers.

Induced and autonomous switchers in the non-student reminder group did not perform (in terms of optimal choice or switching frequency) significantly differently from the group of autonomous switchers in the control group. This suggests that the positive and significant effect of the reminder within the entire non-student sample primarily stems from reminder's efficacy in reducing the proportion of inactive non-students. On the other hand, the reminder had a positive and significant effect on students by improving choices of those who are already autonomous switchers (see Appendix C6 for detailed analysis).

Thus, in contrast with Snowberg & Yariv (2021) who reported population-level shifts, we identified a particular subgroup of participants, those who never would have switched if not prompted, who were overrepresented among non-students (compared to students) and whose behaviour affected the overall performance results of non-students.

## 4 Discussion

The present study shows that, overall, non-students made poorer financial decisions than students. In the control condition non-students switched less frequently, made less optimal switching choices, and displayed a higher level of inertia than students.

Notwithstanding, the reminder had a positive and significant effect on switching frequency and optimal choice for both students and non-students. Although the findings on the effectiveness of the reminder are robust and qualitatively similar across the two samples, the magnitude of the effect differs across the two groups. This result is consistent with previous findings examining differences between students and non-students in lab experiments in policy-applied economic research (Ferré, Engel & Gsottbauer, 2023; Peth & Mußhoff, 2020). It also relates closely to findings by Barr et al. (2023) regarding the generalisability of laboratory results. The authors found a stronger treatment effect on non-students compared to students in the laboratory experiment due to non-students underperformance in the control treatment (random). We contribute to this literature by identifying a specific subgroup of participants, that was over-represented within the non-student sample, and that affected the overall effectiveness of the treatment. In our study, the effectiveness of the reminder was significantly stronger in the non-student sample due to its higher proportion of inactive participants (as evidenced in the control condition; 45.7 % vs. 12.0 % in student sample) who were induced to switch by the reminder.

Indeed, the mechanism through which the informative reminder affected participants differed between students and non-students. In the non-student sample, performance improved because otherwise inactive individuals were induced to switch, and the resulting sample of switchers (both induced and autonomous) performed as well as active switchers in the control condition (autonomous only). However, those non-students who were already active were not significantly affected by the informative reminder, neither in making more optimal choices nor in switching more frequently. On the contrary, in the student sample, the reminder improved performance of active students in terms of optimal choice and switching frequency. In this case, the significant effect of the reminder did not result from a substantial reduction in inactive participants in the reminder condition, possibly due to a floor effect. Instead, it stemmed from the direct impact on switching frequency and optimal decisions.

In contrast with Snowberg & Yariv, (2021), who reported only population-level shifts, we found that a small group of people with extreme behaviour affected the overall performance of the non-student sample. Specifically, inactive subjects — those who did not make any switching decisions in the control condition — were overrepresented among non-students. They were responsible for the low average performance of non-students and their switching behaviour was notably positively affected by the informative message. Moreover, inactive individuals performed more poorly in all cognitive tasks, displayed higher levels of risk aversion, and demonstrated greater impatience. As the switching process necessitated participants to engage in multiple actions while participating in the counting task, their capacity for multitasking (Buser & Peter, 2012) may have played a crucial role in making switching decisions. The need of multitasking might have posed challenges for some participants, especially older ones (Todorov et al., 2014), potentially impacting their engagement in the switching process.

Given the nature of the experimental tasks in this study, our findings could extend to other experimental contexts where attention and cognitive ability are required in order to perform well. A student sample would be sufficient to provide evidence of the effectiveness of a reminder message intervention and would therefore allow for generalizable inferences about behaviour, at least in terms of the direction and statistical significance of the effect. However, experimental evidence based on students should be treated with caution when certain sample characteristics or preferences, which are anticipated to influence the outcome of interest, are underrepresented within the student sample.

These findings raise a red flag on the use of students as a sample for experiments that seek to produce generalizable results and focus on the behaviour of specific subgroups, such as those displaying greater degree of suboptimal behaviour (e.g. ‘inertia’ in the present study) and which may be underrepresented in student samples. For example, as shown in this study, age is positively correlated with inertia. Therefore, the level of inertia of a student sample, that is made by young participants by construction, cannot be representative.

Although the non-student sample has been recruited aiming for a representative distribution with respect to gender, age, and educational level, some discrepancies were observed compared to the respective nationally representative quotas. This sample might also be affected by a potential selection bias, particularly because non-student participants had to commute to an environment, the lab premises of the university, which was probably distant from their everyday-life surroundings. While the experiment might have attracted more “motivated” individuals, the reasonably high show-up fee should have weakened this bias. In our regression analysis, we attempted to address these issues by controlling for different socioeconomic and other confounding factors that could potentially influence financial behaviour and response to the treatment.

Finally, while we selected three countries from different parts of Europe (i.e. Central Europe, Mediterranean and Eastern Europe) to capture the geographical and cultural variety of Europe, we acknowledge that the findings may not be generalizable to other European countries which are culturally or geographically distant from these three. Overall, we found no country effect in almost all the main outcomes of this study (except Polish students who, compared to Spanish students, made more optimal decisions) after controlling for country-specific characteristics. This does not mean that there were no country differences per se. For instance, financial literacy and risk-taking in Spain were significantly lower as compared to Poland and Germany. German participants were more patient (time preference measure) while Polish participants gave fewer wrong answers to the control questions. However, as the focus of this paper was to compare the effectiveness of the reminder treatment between students and non-students across all countries, we used this information only to control for country-specific characteristics and isolate the treatment effect.

The main policy implication of our results is that a simple behavioural intervention, like an informative reminder, can make a significant difference in the behaviour of those who do not exhibit “good” financial behaviour to start with. These are typically consumers with low level of education and financial literacy. This is encouraging for policies aimed at consumers among this specific subgroup, who are trying to navigate the intricacies of the retail financial market. These findings would have not emerged if we had run the study only with students.

However, while a student sample may indeed not adequately capture the behaviour of specific subgroups among non-students, it can nevertheless be a convenient sample when extremes are not important and socio-demographic characteristics are less relevant.

## 5 Conclusion

The question whether a student sample can produce externally valid experimental results continues to pervade research in experimental economics and psychology. This study contributes to the discussion by comparing student and non-student participants’ financial behaviour and response to an *informative* reminder in an experimental setting. Results show that, indeed, there is a difference in behaviour between students and non-students. Students perform better overall, both before and after the intervention. In addition, while the intervention does enhance the performance of participants in both samples, its efficacy is significantly more pronounced on non-students. Many non-students did not switch financial service providers in the control group, but they were successfully prompted to switch by the reminder in the treatment group. On the other hand, *active* non-students (those who switched at least once) showed the same performance as students. Differences are, thus, explained by a subgroup of non-students, the *non-switchers*.

This study supports the view that experimental evidence on convenience samples (such as students) can be generalized, but with caution. It is very likely that within the general population there are subgroups of people —the ‘tails’ — who behave differently from the average. In contrast, the variance in behaviour observed in students’ sample is comparatively smaller. Consequently, when subgroups within the general population are expected to exist and they are relevant to the research question, experimental evidence derived from students may offer only partial insights.

## CRediT authorship contribution statement

**Antonios Proestakis:** Writing – review & editing, Writing – original draft, Visualization, Software, Resources, Methodology, Investigation, Formal analysis, Data curation, Conceptualization. **Ginevra Marandola:** Writing – review & editing, Writing – original draft, Visualization, Software, Resources, Project administration, Methodology, Investigation, Formal analysis, Data curation, Conceptualization. **Joana S. Lourenço:** Writing – review & editing, Writing – original draft, Software, Resources, Project administration, Methodology, Investigation, Funding acquisition, Formal analysis, Conceptualization. **René van Bavel:** Writing – review & editing, Writing – original draft, Resources, Investigation, Funding acquisition.

## Data Availability

Data will be made available on request.

## Appendix Supplementary materials

Image, application 1

## Footnotes

1 The Ethics Committee of Loyola University Andalusia, Spain granted ethical approval.

2 We calibrated the matrix so that it would take on average 40 seconds to solve one, with the aim to ensure that almost all participants could complete at least 24 matrices/months, as the informative reminders were placed every 12 months. On average students solved 32 matrices and the general population 28. 19 % of the students solved 40 matrices and 6 % of the general population solved the 40 matrices.

3 Exchange rates: Germany: 1 euro=125 points; Spain: 1 euro=142 points; Poland: 1 zloty=55 points. Calculation done using consumer price levels in 2017 data (Eurostat, 2018). The final payment for the general population was about 3 times higher than that paid to students as a result of the higher recruitment fee for the general population. This is due to the fact that the two populations have a different opportunity cost for participating (Anderson et al. 2013). The recruitment fee was different across countries and determined by the recruitment company entity responsible to bring the general population sample into the lab. More details in Appendix A2.

4 For each of the student and non-student samples, a comparison of the two experimental groups with regard to socio-demographics, behavioural (i.e. impatience/time preferences and risk aversion) and financial literacy measures can be found in Appendix C1: Balance tests for each of the samples.

5 The distribution of socio-demographic and behavioural characteristics between treatments is available in Appendix C,.

6 While this might appear slightly counter intuitive at first glance, it can be understood by considering that those who were faster were under less time pressure to solve all the matrices in counting task within the allocated time, and the fact that very slow participants did not reach the second (optimal) switching point. Additionally, it is worth noting that being fast can be seen as a proxy for cognitive ability and familiarity with the task, which also helps explains higher performance.

7 The change in trend for non-students at Month 28 might be explained by the fact that less than 50 % of the participants were able to solve more than 28 matrices. Therefore, behaviour after that month is affected by self-selection.

8 We specify random effects at the subject level-two (level-one is the within subject repeated measurements). We defined six (sub-)periods in which the experimental conditions (i.e. market conditions and annual statement exposure) were identical for all subjects and we incorporated the “subperiods” into the random part of the model. The rationale behind this model is that period-specific random factors such as the changes in the market conditions and subjects’ exposure in the annual statements had significant systematic effects on all subjects. We fit a two-way crossed-effects model (see appendix C4 for alternative models), with the subject effects υi being crossed with the periods’ effects ui as follows: $Opt_{ij}=\beta_{0}+\beta_{1}IV_{1}+\ldots +\beta_{n}IV_{n}+u_{i}+\upsilon_{j}+\varepsilon_{ij}$ for the i=1,..,40 months and j=1,…,857 subjects and $u_{i}∼N\left(0,\sigma_{u}^{2}\right)$d*;*$\upsilon_{i}∼N\left(0,\sigma_{\upsilon }^{2}\right)$*;*$\varepsilon_{i}∼N\left(0,\sigma_{\varepsilon }^{2}\right)$*,* all independent. *Opt_ij_* is the dependent variable of the model, β_0_ is the intercept and β_1_…β_n_ the coefficients of the IV_1_…IV_n_ independent variables of the fixed part of the model.

9 Analysis confirms that the differences identified in the control-periods are due to the non-students’ sample (*Reminder*, p < 0.01) and not the student sample (i.e. *Reminder+rem_stud*, n.s.). Crucially, simply comparing the performance of participants after AS1 without accounting for these differences in the control-periods would have yield different results and erroneously suggested that the positive effect of the reminder on optimal choices was not present for both samples. The use of an experimental design that comprised the collection of a baseline (i.e. performance in control periods) for all participants represents therefore an advantage of the present study. It allowed for difference-in-difference analysis and thus overcoming the observed structural differences between the two samples.
