# Supplementary material for: Testing a policy intervention in the lab: differences between students and non-students in switching bank accounts
Source: J Behav Exp Econ. 2024 Aug;111:None. doi: 10.1016/j.socec.2024.102220 (PMC11195910; doi:10.1016/j.socec.2024.102220)
Supplement: Supplementary file 1 [file mmc1.docx]

**appendices**

**Appendix A: Additional information on the sample**

*Appendix A1: Further information about the age composition of the non-student sample in the three countries*

Recruitment of the sample relied on nationally representative quotas for age in each country. For Germany, the Federal Statistical Office (Destatis) projection data for 2019 indicates that the population was composed by 49.0% adults aged 25-60, of which 39.0% aged 25-39, 41.4% aged 40-54, and 19.6% aged 55-60. In our sample, these figures were 38.6%, 42.8%, and 18.7%, respectively. For Spain, the Spanish Statistical Office (INE) data for July 2018 shows that the population was composed by 50.2% adults aged 25-59, of which 37.7% aged 25-39, 48.5% aged 40-54, and 13.8% aged 55-59. In our sample, these figures were 37.8%, 47.1%, and 15.1%, respectively. For Poland, Statistics Poland data for 2016 shows that the population was composed by 50.7% adults aged 25-59, of which 47.1% aged 25-39, 38.3% aged 40-54, and 14.6% aged 55-59. In our sample, these figures were 56.2%, 27.2%, and 16.7% respectively. Recruitment in Poland was done through the lab’s existing recruitment system for non-student participants, which led to slightly higher variation in the quotas due the size of the available database.

*Appendix A2: Additional details on show-up fee*

Testing took place in three university labs: Munich Experimental Laboratory for Economic and Social Sciences (Melessa) at the Ludwig Maximilian University of Munich in Germany, the experimental Economics Lab at the University of Warsaw in Poland and the Laboratorio de Economía Experimental (LEE) at the University Jaume I of Castellón, Spain. Non-students participants received an additional fixed compensation for coming to the lab due to higher opportunity cost of their time relative to the student sample. This show-up fee was €30 in Germany, €25 in Spain and €13.8 (60 Polish złoty) in Poland. It took into consideration differences in purchasing power across countries, as well as guidance from recruitment experts at the marketing research company and university labs. For the non-student sample, higher opportunity cost of time was compounded by the fact that a time commitment of over an hour outside of the working journey was required and subjects needed to incur additional costs (travel and time) to purposely travel to the university campus for the experiment. The show-up fee was mentioned to non-students participants during recruitment.

*Appendix A3: Additional details on educational level categorisation*

*No/primary education* is the proportion of participants with no or primary education. *Secondary education* is the proportion of participants with secondary education. For students there was some noise in the answers as some students who were still enrolled in the undergraduate or master courses declared that their highest level of education was the one in which they were enrolled, while others declared that their highest level of education was the one already obtained. To harmonise the answers we recoded the level of education of students based on answers and age. For students we assign secondary education to every student below 23.

*Undergraduate* is the proportion of participants with undergraduate education. We assign undergraduate to all those who declare to have undergraduate and are above 22. *Master/PhD* is the proportion of participants with Master or Ph.D. We give master or Ph.D. to all those students who declare to have this level of education and are above 23.

**Appendix B: Further information about the experimental procedure, details regarding the experimental environment, and materials**

*B1:Overview of Participants’ earnings and Bank accounts*

*Earnings*

The experiment comprised 40 periods, designated as “months”. In each month, participants had to count all the "1s" in the (12 x 15) matrix displayed on the screen, and earned 35 points every time they gave the correct answer. Earnings in both their current account, and their savings account were visible on the screen in each month and were calculated summarized next. The amount in the current account equalled the sum of gains from performance in the counting task (up to the previous months) minus the monthly current account fee. If switching had occurred, a fee was also deducted from the current account balance. The amount in the savings account equalled an initial endowment of 350 points plus the interest rates on this amount (up to the previous months).

*Bank accounts:*

All participants were assigned the “Triangle” contract (teaser contract, with an initially higher interest rate; Figure B1) by default at the start of the experiment. This generated inertia towards switching, since “Triangle” was the optimal contract for the first 10 months. Once the teaser period ended, “Square” was the optimal choice. After that, once their current account balance was at least 388 points (i.e. by Month 20 if their behavior had been optimal), “Trapezium” became the optimal choice.

Table B1 summarizes the relevant features of the different contracts, as well as the optimal choice strategy.

**Figure B1: “Triangle” contract**


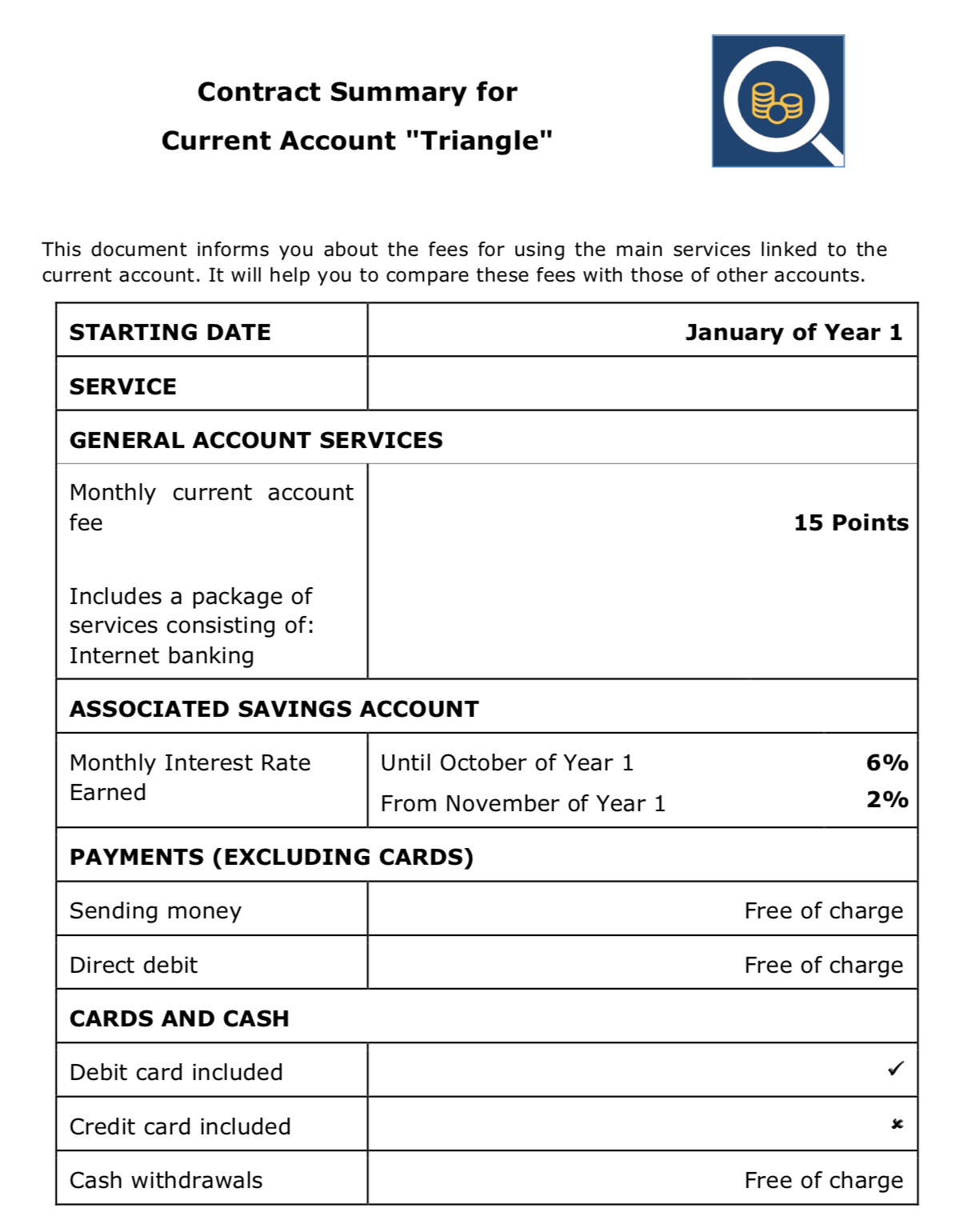


*Note.-* English translation of the “Triangle” account contract as presented to participants (original versions in German, Polish, and Spanish are available upon request). The six contracts differed across three features (starting date, monthly current account fee, and monthly interest rate earned) as summarized in Table B1

**Table B1: Summary of contract features**

| **Contract label** | **Monthly fee for current account** | **Monthly interest rate for savings account** | **Contract availability** | **Optimal choice strategy of contracts** |
| --- | --- | --- | --- | --- |
| Triangle | 15 points | 6% until October Year 1  2% after October Year 1 | January of Year 1 | Jan to Oct Year 1  (Month 1- 10) |
| Square | 15 points if balance in current account is below or equal to 325 points  1 point if balance is above 325 points | 3% | January of Year 1 | Nov Year 1 to July Year 2  (Month 11 - 19) |
| Trapezium | 1 point | 4% | Once balance in current account is higher than 388 points | Aug Year 2 to April Year 4 (End)  (Month 20 - 40) |
| Circle | 15 points | 3% | January of Year 1 | None (distractor contract)^^[[1]](#footnote-1)^^ |
| Rectangle | 15 points | 2.5% | January of Year 1 | None (distractor contract) |
| Hexagon | 16 points | 6% until October Year 1  2% after October Year 1 | January of Year 1 | None (distractor contract) |

**B2: Experimental procedure and participants’ instructions**

Participants received a first set of instructions indicating that the study had two parts. In the beginning of Part 2 they would be assigned a current account contract, which had an associated savings account. This was one of six possible current/savings account contracts. Participants were told they could switch contracts in Part 2. In Part 1 of the study, participants could consult paper versions of the six contracts for 5 minutes. Contracts were removed after this period, and no notes were allowed. Each contract was labelled as a geometric shape, was explained in one page, and had a format analogous to the Fee Information Document used by banks (Appendix B1).

After Part 1, participants received a second set of instructions explaining that they would earn points for performing the counting task, with 25 minutes available to solve up to 40 matrices . Participants were also told how they could check the electronic format of the six contracts, and that they could switch to an alternative contract at any point. To ensure participants understood the experiment, they had to answer comprehension questions.

Participants then completed Part 2 of the study, comprising the counting and switching task, for 25 minutes. The time limit was set to create pressure and ensure that shopping around and switching had an opportunity cost. The counting task, required participants to count all the "1s" in the (12 x 15) matrix displayed on the screen. Participants were allowed to move to the next month/matrix by giving the correct answer (with an error tolerance of +/- 1), or else 100 incorrect ones (so that it was more costly time-wise for a participant to proceed to the next month by giving incorrect random answers rather than solving the matrices).

Participants could shop around and/or switch at any point during the experiment and as many times they wished. However, each time they switched participants incurred a small switching fee (10 points), as well as an opportunity cost (i.e. time away from the incentivized counting task) and hassle. Finally, participants received an annual bank statement every 12 months. In the treatment group, the bank statements also included a salient reminder message (at the top, in bold text and different color) encouraging switching. The message was created using the feedback on switching collected from participants during the pilot, and included three with positive and one with negative connotation for balance. They included mention to main features of the contracts and highlighted the trade-off between switching and performing the counting task.

After Part 2, participants completed a risk aversion task, followed by the final questionnaire. At the end of the experiment, participants were debriefed and paid in private.

**Experimental instructions**

| **INSTRUCTIONS**  Welcome to this study and thank you for your participation! Please do not talk to the other participants from now on. These instructions are a detailed description of the procedures we will follow.  You can earn money during the study depending on the choices you will make. You will be paid privately and in cash at the end of the study.  During the study you are not allowed to communicate with the other participants. We also ask you to switch off your mobile phone now. If you need assistance at any time, please raise your hand and remain seated: someone will come to your desk to answer it.  The study is divided into **Part 1 and Part 2.** Your final earnings will depend on your effort and decisions in both Parts. Your earnings will be expressed in points. The points will be converted in euros at the end of the study. The conversion rate is 125 points = €1 (*Germany*).  As we proceed with the instructions, you will be asked to answer 3 questions designed to verify if you understood the instructions. |
| --- |
| **-Part 1-**  **The contracts**  At the beginning of the study you will be assigned a current account contract. Your earnings from Part 2 will be paid into **your current account**. This current account has an associated **savings account**.  You will be endowed with 350 points in your savings account at the start, and in each month you will earn interests on the amount you have on your saving account, which will be added to this account. For example, if in month 1 you have 100 points and your interest rate is 5%, in month 2 you will have 105 points, in month 3 you will have 110.25 points, in month 4 you will have 115.76 and so on until the end of the period. The amount of points that you have in your savings account will be visible on the screen in every month.  There are six different current account contracts, each with an associated savings account. You will be provided with the summary of each of these contracts. This 1-page document summarises the fees and services associated with the current account and the interests on the savings account. If there is a monthly fee associated with the current account this will be subtracted from the earnings accumulated in your current account in Part 2. The interests produced by your initial endowment of 350 points will be accumulated on your saving account. The summaries will be handed out to you (in paper format).  As mentioned earlier, you will be automatically assigned to a current account contract and its associated saving account at the start of Part 2. Read all the contracts carefully. In Part 2 you will have the possibility to keep that contract or to check again the other available contracts and switch to one of them.  You will now be given 5 minutes to look at the contracts before we take the documents back. |
| **-Part 2 -**  In Part 2 you are asked to perform a task involving counting all the "1" digits in a table displayed on the screen. You will be presented several tables, one at a time, and each table is associated with a given month and year. Only when you get the right number of 1s in the table, you will be able to move to the next one. You will earn 35 points for each correct completed table you get.  **The task**  On the screen you will see a table with digits ranging from "1" to “9”. You will be asked to count all the "1" digits in the table. Any entry falling within a range of -1 to +1 from the correct number will be considered correct. If you indicate a wrong number (outside the acceptable margin of error) you will be able to try again.  You can move on to the next month by clicking "Continue" once you enter a count that is correct. If you make 100 incorrect attempts you will be automatically moved to the next month without earning any points from this table.  Below you see an example of a monthly screen.  *Screenshot: Monthly screen (see Step 1 in Figure 2)*    As shown in the image, the solution for this table is 37. Your answer will be considered correct if you input 37, but also if you input 36 or 38.  You will start Part 2 in January of Year 1 and after you correctly count the "1s" in the first table you will be able to continue to February of Year 1 and so forth.  Part 2 has the duration of 25 minutes, and the programme will stop automatically at the end of this time. You will see a timer at the top of the screen. You will be able to perform the counting task as many times as you manage in the time given for Part 2. However, there is a maximum amount of tables available, which is 40 (equivalent to 40 months). If you complete the 40 tables you will not be able to perform the task again, while you will be able to do the other activities in this study until the time elapses.  Your earnings from the counting task will be paid into your current account in the next month. If there is a monthly fee associated with the current account this will be subtracted from the earnings accumulated in your current account. For instance, if you find the correct answer in January Year 1, 35 points will be added in your current account in February. If your monthly fee is 12 this amount will be subtracted from your current account in February. As a result in the beginning of February Year 1 you will see 23 (=35-12) points added to your current account. The amount of points that you have in your current account and in the associated savings account will be visible on the screen in every month.  **The contracts**  There are six different current account contracts (each with an associated savings account) and you have been given 5 minutes to check these contracts in Part 1.  You will be automatically assigned to a current account contract at the start of Part 2. You will see the contract assigned to you on the first screen. It is your choice to keep it, or to check again the alternative contracts and switch to one of these during Part 2.  A mailbox is presented on the screen in every month. You can check the different contracts and switch by clicking on the mailbox and then clicking on the email with the title "Check your bank account". There will be other emails in the mailbox, and you can read these at any point if you like.  Below you see an example of the mailbox.  *Screenshot: Mailbox (see Step 2 in Figure 2)*    As you can see below, once you open the email with the title "Check your bank account" you will be able to:   - Check information about alternative accounts: you can click on any of the contracts in order to read the information concerning that contract (services, interest rate, current account fee etc. etc.); - Switch to an alternative account: you can click on the switch button in order to switch to a different contract of your choosing; - Go back to the monthly task: you can click on the return to the task button to go back to the monthly task if you decide not to switch.   Below you see an example.  *Screenshot: Contracts screen (see Step 3 in Figure 2)*    If you click on the "Switch" button, you will be able to select the contract you would like to switch to and then return to the task by clicking on "Confirm and Return to task". If you change your mind once you're on this screen and decide not to switch, you will be able to do so by clicking on "Don’t switch and Return to task".  You can go back to the counting task at any moment. If you switch, the conditions of the new contract will apply to you from the following month and for the rest of the study if you don't switch again.  You can switch as many times you like and at any moment in time.  The timer will not stop while you go to the mailbox, when you look at the emails, or when you take actions in order to look at the contracts or to switch. This means that the time you spend on other activities is taken from the time available for performing the counting task.  **The annual statement**  Starting in January of Year 2, and in every subsequent January, you will receive an annual statement. You will be given a minimum of 5 seconds to look at it. After 5 seconds the button "Continue" will appear on the screen and you can click on it to move to the next month when you're ready. Please read the annual statement carefully as it will contain relevant information for you.  *Screenshot: Annual Statement (see Step 4 in Figure 2)*  **The payment**  Your total earnings for Part 2 is calculated as follows:  Total points in Current Account + Total points in Savings Account  At the end of the study the total amount of Points you have earned will be converted to Euros at the following rate:  **125 points = 1 Euro**  Your earnings from the study will be paid to you privately and in cash at the end.  **The questionnaire**  After you have completed the study you will be asked to fill-in a short questionnaire. The answers you give are important for our scientific analysis so please fill it in truthfully. |

**B5: Multiple-choice questions checking participants’ understanding of the experimental procedure and payoff system**

1. Please answer the following 2 questions, to ensure you fully understand the instructions.

2. If you have switched in month 12 and you are in month 16, can you switch again?

1. Yes [correct]
2. No

3. If the solution to the tables is 30 and you wrote down 31 as the correct answer, will your answer be considered correct?

1. Yes [correct]
2. No

**Appendix C: Further data and analysis**

**C1: Balance tests for each of the samples**

For each of the sample of students and non-students, balance t-tests were carried out between baseline and reminder groups for the socio-demographic variables, behavioural and the two measures associated with the experimental task. The groups were generally well balanced on all variables, with a few exceptions. For students, the proportion of males was higher in the baseline (0.57) than in the reminder (0.42) group (p=0.004). Additionally, financial literacy was higher for those in the reminder (2.42) than those in the baseline (2.22) group, but the difference was only marginally significant (p=0.051). For non-students, those in the reminder group required fewer attempts (2.46) to correctly answer the three multiple-choice questions about the instructions than those in the baseline (3.03) group (p<0.001); there were no other statistically significant differences. These variables have been included as covariates in the regression analyses to control for observed differences between groups.

**Table C1a: Balance tests for each of the samples**

|  | **Students** | | | | | | | **Non-students** | | | | | | |
| --- | --- | --- | --- | --- | --- | --- | --- | --- | --- | --- | --- | --- | --- | --- |
|  | **Baseline** | |  | **Reminder** | |  |  | **Baseline** | |  | **Reminder** | |  |  |
|  | (n = 176) | |  | (n = 181) | |  |  | (n = 245) | |  | (n = 255) | |  |  |
|  | Mean | Std. dev. |  | Mean | Std. dev. |  | *p-*Value | Mean | Std. dev. |  | Mean | Std. dev. |  | *p-*Value |
| Age (years) | 21.16 | 2.22 |  | 21.4 | 2.07 |  | n.s. | 41.97 | 11.07 |  | 40.99 | 10.55 |  | n.s. |
| Male (proportion) | .57 | .5 |  | .42 | .49 |  | 0.004 | 0.50 | 0.50 |  | .49 | .5 |  | n.s. |
| Education | 2.5 | .75 |  | 2.55 | .72 |  | n.s. | 2.67 | 0.94 |  | 2.7 | .93 |  | n.s. |
| Financial liter. (0‒3) | 2.25 | .92 |  | 2.42 | .82 |  | 0.051 | 2.33 | 0.81 |  | 2.41 | .79 |  | n.s. |
| Very impatient (0‒1) | .1 | .3 |  | .1 | .31 |  | n.s. | 0.11 | 0.32 |  | .11 | .31 |  | n.s. |
| Risk aversion (0‒1) | .52 | .5 |  | .5 | .5 |  | n.s. | 0.55 | 0.50 |  | .48 | .5 |  | n.s. |
| Wrong Ans. (0‒4) | 2.13 | 1.44 |  | 2.29 | 1.45 |  | n.s. | 3.03 | 1.34 |  | 2.44 | 1.64 |  | 0.000 |
| No. Months (1‒40) | 32.43 | 6.65 |  | 31.42 | 6.25 |  | n.s. | 27.71 | 6.86 |  | 27.89 | 7.1 |  | n.s. |

*Age* is the age of participant in years. *Male* is the proportion of men in the sample. *Education=1* is no or primary education. *Education=2* is secondary education. For students we assign secondary education to every student below 23. *Education=3* is undergraduate education. We assign undergraduate to all those who declare to have undergraduate and are above 22. *Education=4* is Master or Ph.D. We give master or Ph.D. to all those students who declare to have this level of education and are above 23. *Fin. Literacy* goes from 0 to 3 that is the number of correct answers to financial literacy questions; *Very impatient* are those who never change the present for a future higher compensation, dummy equal to 1; *Risk Aversion*: all those people who have investment<=40, dummy equal 1 for risk averse participants. *Wrong Answers,* the variable is 0 if the participant gets all the answers at the first trial, 1 if 1 additional attempt is needed, 2 for two attempts, 3 for 3 attempts, 4 if at the third attempt they were still wrong and therefore they need to answer again after having received the correct answers.

**Table C2b: Descriptive statistics for each of the samples in Germany**

| **GERMANY** | **Students** | | | | | | | **Non-students** | | | | | |
| --- | --- | --- | --- | --- | --- | --- | --- | --- | --- | --- | --- | --- | --- |
|  | **Baseline** | |  | **Reminder** | |  | **Baseline** | |  | **Reminder** | |  |  |
|  | (n = 57) | |  | (n = 65) | |  | (n = 80) | |  | (n = 86) | |  |  |
|  | Mean | Std. dev. |  | Mean | Std. dev. |  | Mean | Std. dev. |  | Mean | Std. dev. |  |  |
| Age (years) | 20.91 | 2.24 |  | 22.12 | 2.43 |  | 44.27 | 10.59 |  | 42.16 | 9.93 |  |  |
| Male (proportion) | .51 | .5 |  | .38 | .49 |  | .51 | .5 |  | .49 | .5 |  |  |
| Education | 2.49 | .78 |  | 2.82 | .79 |  | 2.44 | .76 |  | 2.71 | .85 |  |  |
| Financial literacy (0‒3) | 2.09 | 1.04 |  | 2.51 | .75 |  | 2.6 | .59 |  | 2.49 | .82 |  |  |
| Very impatient (0‒1) | .07 | .26 |  | .09 | .29 |  | .05 | .22 |  | .02 | .15 |  |  |
| Risk aversion (0‒1) | .47 | .5 |  | .45 | .5 |  | .45 | .5 |  | .44 | .5 |  |  |
| Wrong Answers (0‒4) | 2.19 | 1.41 |  | 2.08 | 1.47 |  | 2.99 | 1.31 |  | 2.7 | 1.47 |  |  |
| No. Months (1‒40) | 31.33 | 5.99 |  | 32.85 | 6.35 |  | 27.81 | 6.86 |  | 27.74 | 6.01 |  |  |

**Table C3c: Descriptive statistics for each of the samples in Poland**

| **POLAND** | **Students** | | | | | | | **Non-students** | | | | | |
| --- | --- | --- | --- | --- | --- | --- | --- | --- | --- | --- | --- | --- | --- |
|  | **Baseline** | |  | **Reminder** | |  | **Baseline** | |  | **Reminder** | |  |  |
|  | (n = 59) | |  | (n = 57) | |  | (n = 79) | |  | (n = 83) | |  |  |
|  | Mean | Std. dev. |  | Mean | Std. dev. |  | Mean | Std. dev. |  | Mean | Std. dev. |  |  |
| Age (years) | 21.51 | 2 |  | 20.88 | 1.51 |  | 39.82 | 11.96 |  | 38.23 | 11.7 |  |  |
| Male (proportion) | .75 | .44 |  | .46 | .5 |  | .47 | .5 |  | .48 | .5 |  |  |
| Education | 2.63 | .79 |  | 2.39 | .53 |  | 3.37 | .82 |  | 3.19 | .89 |  |  |
| Financial literacy (0‒3) | 2.73 | .64 |  | 2.68 | .69 |  | 2.44 | .76 |  | 2.48 | .72 |  |  |
| Very impatient (0‒1) | .12 | .33 |  | .16 | .37 |  | .14 | .35 |  | .12 | .33 |  |  |
| Risk aversion (0‒1) | .49 | .5 |  | .46 | .5 |  | .57 | .5 |  | .41 | .49 |  |  |
| Wrong Answers (0‒4) | 1.86 | 1.36 |  | 2.28 | 1.54 |  | 3.08 | 1.37 |  | 1.55 | 1.82 |  |  |
| No. Months (1‒40) | 35.1 | 5.86 |  | 31.23 | 6.71 |  | 27.48 | 7.28 |  | 28.63 | 8.03 |  |  |

**Table C4d: Descriptive statistics for each of the samples in Spain**

| **SPAIN** | **Students** | | | | | | | **Non-students** | | | | | |
| --- | --- | --- | --- | --- | --- | --- | --- | --- | --- | --- | --- | --- | --- |
|  | **Baseline** | |  | **Reminder** | |  | **Baseline** | |  | **Reminder** | |  |  |
|  | (n = 60) | |  | (n = 59) | |  | (n = 86) | |  | (n = 86) | |  |  |
|  | Mean | Std. dev. |  | Mean | Std. dev. |  | Mean | Std. dev. |  | Mean | Std. dev. |  |  |
| Age (years) | 21.07 | 2.39 |  | 21.1 | 1.9 |  | 41.8 | 10.36 |  | 42.49 | 9.51 |  |  |
| Male (proportion) | .47 | .5 |  | .42 | .5 |  | .51 | .5 |  | .51 | .5 |  |  |
| Education | 2.38 | .67 |  | 2.42 | .72 |  | 2.23 | .82 |  | 2.22 | .79 |  |  |
| Financial literacy (0‒3) | 1.93 | .84 |  | 2.07 | .89 |  | 1.99 | .9 |  | 2.27 | .82 |  |  |
| Very impatient (0‒1) | .12 | .32 |  | .07 | .25 |  | .15 | .36 |  | .17 | .38 |  |  |
| Risk aversion (0‒1) | .58 | .5 |  | .61 | .49 |  | .62 | .49 |  | .58 | .5 |  |  |
| Wrong Answers (0‒4) | 2.32 | 1.52 |  | 2.54 | 1.32 |  | 3.02 | 1.35 |  | 3.03 | 1.24 |  |  |
| No. Months (1‒40) | 30.83 | 7.24 |  | 30.03 | 5.38 |  | 27.83 | 6.52 |  | 27.31 | 7.18 |  |  |

**C2: Trade-off between switching and counting task.**

As shown in the table below, there is a positive correlation between switching frequency and performance in the counting task for non-students (column 1), this is not the case for students (column 2) and for active non-students (column 3). The coefficient on switching in column 1 is basically capturing the active group, confirming that active participants switch more and are faster in solving matrices. In column 3, the coefficient on the reminder is negative and significant. Active participants in the reminder treatment, are slower in the counting task than active participants in the control. This confirms that the reminder induces switching in those participants that are less performing, increasing heterogeneity in the active group.

**Table C5: the effect of switching on the distractor task**

|  | (1) | (2) | (3) | (4) |
| --- | --- | --- | --- | --- |
|  | Number of months | Number of months | Number of months | Number of months |
| Switching | .046*** | 0 | -.009 | -.008 |
|  | (.011) | (.011) | (.015) | (.013) |
| Reminder | -.029 | -.029 | -.064** | -.035 |
|  | (.022) | (.021) | (.024) | (.021) |
| Male | -.011 | .006 | -.005 | -.005 |
|  | (.022) | (.022) | (.024) | (.023) |
| Fin. literacy | .025 | .009 | .021 | .013 |
|  | (.014) | (.013) | (.018) | (.013) |
| V. Impatient | .047 | -.081* | .048 | -.084 |
|  | (.038) | (.038) | (.044) | (.046) |
| Risk averse | -.019 | -.05* | -.012 | -.043* |
|  | (.022) | (.021) | (.025) | (.021) |
| Wrong answers | -.026*** | -.018** | -.016* | -.015* |
|  | (.007) | (.007) | (.008) | (.007) |
| _cons | 3.314*** | 3.491*** | 3.433*** | 3.507*** |
|  | (.045) | (.04) | (.058) | (.045) |
| Observations | 500 | 357 | 319 | 317 |
| Pseudo R^2^ | .024 | .018 | .012 | .015 |
| Country Dummies | Yes | Yes | Yes | Yes |
| Year Dummies | Yes | Yes | Yes | Yes |
| *Robust standard errors are in parentheses* | | | | |
| **** p<.005, ** p<.01, * p<.05* | | | | |
|  | | | | |
| This is a Poisson with robust standard error. The dependent variable is the number of months reached by the participant. In columns 1 and 3 the sample is non-students, in columns 2 and 4 the sample is student sample. In column 3 and 4 the analysis is conducted on the sub-sample of active participants (those who switch at least once). *Reminder* is a dummy = 1 the observation comes from the treatment; *Male* is =1 if participant is male; *Fin. Literacy* goes from 0 to 3 that is the number of correct answers to financial literacy questions; *Age* is the age of participant in years. *Very impatient* are those who never change the present for a future higher compensation, dummy equal to 1. *Risk Aversion*: all those people who have investment<=40, dummy equal 1 for risk averse participants. *Wrong Answers,* the variable is 0 if the participant gets all the answers at the first trial, 1 if 1 additional attempt is needed, 2 for two attempts, 3 for 3 attempts, 4 if at the third attempt they were still wrong and therefore they need to answer again after having received the correct answers. *Undergraduate* is the proportion of participants with undergraduate education. *Master/PhD* is the proportion of participants with Master or Ph.D. *#Months* is the number of months (matrices) reached by the participant. We controlled for country dummies. | | | | |

**C3: The effect of reminder on non-students’ Inertia using interaction**

The effects on non-students were heterogeneous. The reminder reduced inertia among female but not male participants, as the linear combination of the coefficient on reminder and its interaction with the coefficient on male (-.47) was not found statistically significant (p-value = 0.208). The reminder reduced inertia of participants who were slower (below the median) in the counting task but not of those who were faster (above the median). Similarly, the linear combination (-.44) of the coefficient on *Reminder* and its interaction with the coefficient on *Number of months* above the median was not statistically significant (p-value = 0.250). The latter result suggests that the effect of the reminder was stronger on those who performed worse in the distractor task, that is, those participants for which the trade-off between switching and solving the matrices was higher.

**Table C3: Determinants of financial decisions for non-students**

|  | (1) | (2) | (3) |
| --- | --- | --- | --- |
|  | Inertia | Inertia | Inertia |
| Reminder | -.997*** | -1.469*** | -1.376*** |
|  | (.258) | (.363) | (.354) |
| Male | -.031 | -.515 | -.011 |
|  | (.27) | (.355) | (.268) |
| RemMale |  | .996* |  |
|  |  | (.526) |  |
| Fin. literacy | -.629*** | -.65*** | -.644*** |
|  | (.159) | (.163) | (.161) |
| Undergraduate | -.645* | -.619 | -.654* |
|  | (.368) | (.379) | (.363) |
| Master/Phd | -.629* | -.57 | -.667* |
|  | (.382) | (.377) | (.373) |
| Age | .04*** | .041*** | .043*** |
|  | (.013) | (.013) | (.013) |
| V. Impatient | -.106 | -.1 | -.117 |
|  | (.401) | (.409) | (.398) |
| Risk averse | .454* | .429 | .447* |
|  | (.269) | (.27) | (.265) |
| Wrong answers | .014 | .004 | .042 |
|  | (.096) | (.096) | (.094) |
| # Months | -.092*** | -.093*** |  |
|  | (.023) | (.023) |  |
| med_month |  |  | -1.471*** |
|  |  |  | (.386) |
| RemMedMonth |  |  | .938* |
|  |  |  | (.52) |
| Germany | -.158 | -.172 | -.168 |
|  | (.327) | (.327) | (.327) |
| Poland | .802** | .772** | .786** |
|  | (.374) | (.365) | (.369) |
| Observations | 339 | 339 | 339 |
| Pseudo R^2^ | .215 | .223 | .209 |
| *Robust standard errors are in parentheses* | | | |
| **** p<.01, ** p<.05, * p<.1* | | | |
| This is logit robust standard errors. The regression only includes participants that are inactive before the reminder. The dependent variable is =1 if the participant never switched. *Reminder* is a dummy = 1 if the observation comes from the treatment; The independent variables  *Male*, *Fin. Literacy*, *Age* , *Very , Risk Aversion*, *Wrong Answers,Undergraduate*, *Master/PhD*, *#Months* as defined in  Table 3. In column (2) we include the interaction between the Reminder and male, i.e. RemMale and in column (3) the variable med_month, which is a dummy taking the value 1 if subject’s tot_month is higher than the sample’s median and its interaction with reminder, RemMedMonth.. | | | |

**C4: Alternative multilevel models and Akaike comparison**

In Table C4, we test different specifications of our basic model of Table 4 (column 2). In the first specification (Model 1) we include two random intercepts in the random part of the model (level 1: repeated measurements, level 2: subjects) without any random slope. Time enters only in the fixed part of the model by including 40 (-1 for avoiding multicollinearity) dummies, one for each month (omitted from the table). In Model 2 we have again a two-level model, but now we also include time in the random part of the model as a random slope. In this model we assume that the effect due to “months” is linear and subject specific. Model 3 is similar to model 2, but instead of “months” we use “period” as a random slope (again linear effect). In both models 2 and 3 we assume an independent covariance structure in the random part u_j_ + v_j*_month_ij_ and u_j_ + v_j*_period_ij_ respectively. Independent covariance structure allows for a distinct variance for each random effect within a random-effects equation and assumes that all covariances are 0. Model 4 is identical to model 3 but we assume an unstructured covariance structure in the random part u_j_ + v_j*_period_ij_, which allows for all variances and covariances to be distinct. By specifying covariance(unstructured) in model 4, we told to our mixed logit model to allow correlation between random effects at the subject level. Finally model 5 is the two-way crosses effect model we illustrated earlier in Table 4. In this model the default covariance structure given by the statistical software is identity, that is all variances are equal and all covariances are 0.

As shown in table C4 the triple interaction in rem_AS_std is negative and significant in all models, indicating that in the reminder treatment (afterAS1) students made less optimal choices as compared to non-students. The main difference between the models is that in all models 2, 3 and 4 (where we include the random slopes in the random part) the effect of reminder (afterAS1) on the students sample, captured by the sum ReminderXas+rem_AS_std is not any more significant, indicating that the reminder did not actually affected students positively at all.

In the bottom of table C4, we also report the AIC and BIC statistics of each model. We can see that from statistical point of view the alternative models are performing even better than our basic model 5. However, we prefer to show the results of model 5 in our main analysis because conceptually the specifications of this model 5 is closer to our experimental design: all subjects were affected systematically by the same period-specific random factors such as the changes in the market conditions and subjects’ exposure in the annual.

Table C4: The effect of reminder on the choice of optimal contract, alternative models

|  | (1) | (2) | (3) | (4) | (5) |
| --- | --- | --- | --- | --- | --- |
|  | Optimal_Contract | Optimal_Contract | Optimal_Contract | Optimal_Contract | Optimal_Contract |
| Reminder | -.548*** | -.674** | -1.019** | -.408** | -.527*** |
|  | (.169) | (.293) | (.423) | (.184) | (.163) |
| afterAS1 | -3.025*** | -2.752*** | -4.055*** | -4.165*** | -1.504 |
|  | (.404) | (.146) | (.211) | (.212) | (1.104) |
| Student | -.421** | -.701** | -1.51*** | -.06 | -.471** |
|  | (.189) | (.328) | (.477) | (.207) | (.186) |
| ReminderXAS | .801*** | .66*** | 1.077*** | 1.156*** | .787*** |
|  | (.108) | (.196) | (.273) | (.273) | (.107) |
| rem_std | .442* | .228 | .469 | .417 | .434* |
|  | (.261) | (.442) | (.642) | (.276) | (.252) |
| AS_std | 1.249*** | .754*** | .59** | .693** | 1.247*** |
|  | (.115) | (.206) | (.285) | (.285) | (.114) |
| rem_AS_std | -.445*** | -.677** | -1.232*** | -1.35*** | -.439*** |
|  | (.158) | (.281) | (.382) | (.381) | (.156) |
| Male | .295** | -.518** | -.823** | -.091 | .291** |
|  | (.12) | (.223) | (.324) | (.136) | (.116) |
| Fin. literacy | .256*** | -.48*** | -.712*** | .001 | .239*** |
|  | (.076) | (.144) | (.208) | (.089) | (.073) |
| V. Impatient | -.166 | .732** | 1.118** | .073 | -.158 |
|  | (.193) | (.367) | (.53) | (.224) | (.186) |
| Risk averse | -.199* | -.125 | -.137 | -.149 | -.183 |
|  | (.119) | (.22) | (.32) | (.134) | (.115) |
| Wrong answers | -.164*** | .018 | .085 | -.118** | -.149*** |
|  | (.041) | (.076) | (.11) | (.046) | (.039) |
| # Months |  | -.118*** | -.163*** | -.007 | .015* |
|  |  | (.017) | (.025) | (.011) | (.009) |
| Germany | .033 | -.306 | -.553 | .01 | .029 |
|  | (.146) | (.269) | (.391) | (.163) | (.14) |
| Poland | .316** | .108 | .09 | .302* | .3** |
|  | (.15) | (.278) | (.404) | (.17) | (.144) |
| Observations | 25293 | 25293 | 25293 | 25293 | 25293 |
| Month Dum. (fixed part) | yes | no | no | no | no |
| Chi^2^ | 4866.863 | 1126.831 | 1344.194 | 1353.759 | 314.374 |
| ll(model) | -11177.85 | -10945.6 | -10173.3 | -9412.85 | -11441.3 |
| AIC | 22463.7 | 21927.18 | 20382.69 | 18863.71 | 22918.65 |
| BIC | 22903.17 | 22073.67 | 20529.17 | 19018.33 | 23065.14 |
| *Standard errors are in parentheses* | | | | | |
| **** p<.01, ** p<.05, * p<.1* | | | | | |
|  | | | | | |

**C5: The effect of the second reminder**

The repetition of the intervention, a second reminder message in month 24, did not have any statistically significant effect in generating by Month 25 an increase in the percentage of participants who switched to the right contract. As shown from the positive coefficient on afterAS2, in Month 25 participants start behaving more optimally as they realize they need to switch to a new contract available as of Month 20-21. Moreover, after Month 25 only faster participants are observed, as the slower ones do not reach that many matrices in the 25 minutes time. However, the improvement in optimal choices does not depend on the reminder message as the coefficient on *ReminderXAS2* is not statistically different from zero. Therefore, in our setting where the first and second reminder are close in terms of time, the repetition of the intervention does not prove to be useful.

**Table C5: Effect of second reminder**

|  |  |  | |
| --- | --- | --- | --- |
|  | (1) | (2) | |
|  | Optimal_Contract | Optimal_Contract | |
| Reminder | -.348*** | -.161 | |
|  | (.124) | (.134) | |
| afterAS1 | -1.385 | -.506 | |
|  | (1.036) | (.745) | |
| afterAS2 | 1.73 | 1.975** | |
|  | (1.31) | (.943) | |
| Student | .434*** | .109 | |
|  | (.119) | (.125) | |
| ReminderXAS | .62*** | .163* | |
|  | (.084) | (.086) | |
| reminderAS2 | -.1 | -.017 | |
|  | (.094) | (.111) | |
| Male | .292** | .243* | |
|  | (.115) | (.124) | |
| Fin. literacy | .235*** | .12 | |
|  | (.073) | (.085) | |
| V. Impatient | -.161 | .038 | |
|  | (.184) | (.215) | |
| Risk averse | -.188* | -.135 | |
|  | (.114) | (.123) | |
| Wrong answers | -.144*** | -.126*** | |
|  | (.039) | (.041) | |
| # Months | .013 | .012 | |
|  | (.009) | (.01) | |
| Germany | .034 | .077 | |
|  | (.139) | (.149) | |
| Poland | .317** | .376** | |
|  | (.143) | (.157) | |
| Observations | 25293 | 19592 | |
| Pseudo R2 | .z | .z | |
| Chi2 | 149.899 | 48.057 | |
| *Standard errors are in parentheses* | | | |
| **** p<.01, ** p<.05, * p<.1* | | | |
|  | | | |

Multilevel (mixed effects) logit regression models on the binary dependent variable “Optimal Contract”, equal to one if the participant holds the best available contract in the current month, 0 otherwise. Column 1 corresponds to the full sample and column 2 to the active sample. *Reminder* is a dummy variable capturing the treatment effect; *AfterAS1* is a dummy indicating the months after the first annual statement (i.e. month >12) and *AfterAS2* is a dummy indicating the months after the second annual statement (i.e. month >24); *ReminderXAS* is the interaction of *AfterAS1* and *Reminder*, *ReminderXAS2* is the interaction of *AfterAS2* and *Reminder*. We control for *Age, Male, Fin. Literacy, Age, Very impatient, Risk Aversion, Wrong Answers, Undergraduate, Master/PhD, Country* and total number of *Months* as defined in

Table 3. The panel dataset is unbalanced due to the fact that not all participants managed to solve all matrices and therefore go through all 40 months Since we have a panel, the total number of observations is the product of the number of subjects (n) and the corresponding number of months for each subject.

**C6: Active Sample: Differences in performance between students and non-students**

## **Figure C6a: Switching frequency for ‘active’ non-students and students**


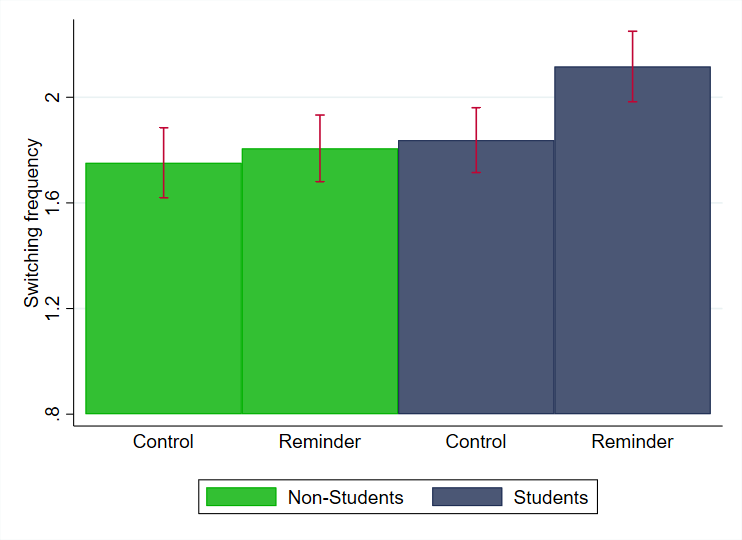


**Table C6a: Switching frequency, full and active samples**

|  | (1) | (2) | (3) | (4) |
| --- | --- | --- | --- | --- |
|  | Switch  (Full) | Switch  (Full) | Switch  (Active) | Switch (Active) |
| Student | .354*** | .397*** | .116*** | .039 |
|  | (.051) | (.08) | (.035) | (.052) |
| Reminder | .234*** | .273*** | .091** | .021 |
|  | (.051) | (.081) | (.035) | (.051) |
| rem_std |  | -.074 |  | .132* |
|  |  | (.102) |  | (.069) |
| Male | .147*** | .144*** | .083** | .086** |
|  | (.052) | (.052) | (.036) | (.036) |
| Fin. literacy | .181*** | .182*** | .057** | .055** |
|  | (.036) | (.036) | (.023) | (.023) |
| V. Impatient | -.151 | -.15 | -.042 | -.046 |
|  | (.094) | (.095) | (.064) | (.064) |
| Risk averse | .014 | .014 | .044 | .044 |
|  | (.05) | (.05) | (.035) | (.035) |
| Wrong answers | -.073*** | -.072*** | -.036*** | -.038*** |
|  | (.016) | (.016) | (.011) | (.011) |
| # Months | .014*** | .014*** | -.002 | -.002 |
|  | (.004) | (.004) | (.003) | (.003) |
| Observations | 857 | 857 | 636 | 636 |
| Pseudo R^2^ | .059 | .059 | .008 | .009 |
| Country Dummies | Yes | Yes | Yes | Yes |
|  | | | | |

Poisson regressions with robust standard error. The dependent variable is the total number of switches or switching frequency. In columns 1 and 2 we observe the full sample (identical to Table 3), in column 3 and 4 the analysis is conducted on the sub-sample of active participants (those who switch at least once). *Reminder* is a dummy ( = 1) if the observation comes from the reminder treatment and student is dummy (=1) if the observation comes from students’ sample. We control for *Male, Fin. Literacy, Very impatient, Risk Aversion, Wrong Answers, total number of months and Country* as defined in Table 3 . In columns 2 and 4 we also include the interaction between Reminder and Student, rem_std. **** p<.01, ** p<.05, * p<.1*

Figure C6a displays switching frequency for participants in the active subsample and indicates that while there are no differences between the control and reminder group of active non-students, there are differences for active students , which is confirmed by regression estimates (rem_std, p =0.057; Table C6a, column 4). This means that non-students who are induced to switch by the reminder, do not switch significantly more often than spontaneous switchers (at least not enough to change the average switching frequency). On the other hand, among students it seems that the reminder makes spontaneous switchers switch more often, increasing the switching frequency above the optimal value of 2.

Figure C6b: Optimal choice path for ‘active’ non-students and students


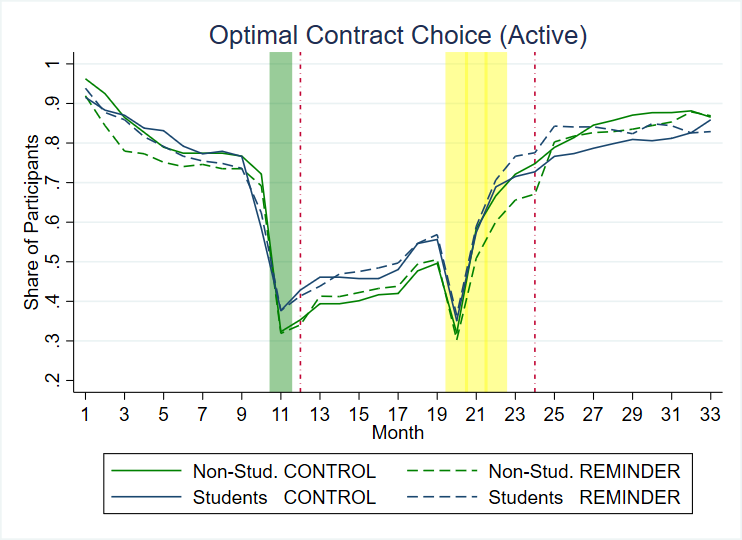


The green and yellow shadow areas represent the optimal switching points. Red dotted lines (Months 12 and 24) indicate the timing of the switching reminder presented to non-students and student participants in the treatment/reminder condition. The green lines represent the share of non-students participants holding the best contract in each month, and the blue ones the share of students. For both non-students and students, the continuous lines represent the control condition, and the dotted lines represent the reminder or treatment condition.

Table C6b: Effect of the reminder on optimal choice

| \|  \| (1) \| (2) \| (3) \| (4) \| \| --- \| --- \| --- \| --- \| --- \| \|  \| *Opt_ij_* \| *Opt_ij_* \| *Opt_ij_* (active) \| *Opt_ij_*  (active) \| \| Reminder \| -.345*** \| -.527*** \| -.16 \| -.285 \| \|  \| (.124) \| (.163) \| (.134) \| (.188) \| \| afterAS1 \| -.947 \| -1.504 \| -.011 \| -.007 \| \|  \| (1.109) \| (1.104) \| (.929) \| (.93) \| \| Student \| .434*** \| -.471** \| .109 \| -.081 \| \|  \| (.119) \| (.186) \| (.125) \| (.199) \| \| ReminderXAS \| .585*** \| .787*** \| .159** \| .073 \| \|  \| (.078) \| (.107) \| (.08) \| (.115) \| \| rem_std \|  \| .434* \|  \| .242 \| \|  \|  \| (.252) \|  \| (.264) \| \| AS_std \|  \| 1.247*** \|  \| -.006 \| \|  \|  \| (.114) \|  \| (.119) \| \| rem_AS_std \|  \| -.439*** \|  \| .176 \| \|  \|  \| (.156) \|  \| (.16) \| \| Male \| .291** \| .291** \| .243* \| .251** \| \|  \| (.115) \| (.116) \| (.124) \| (.124) \| \| Fin. Literacy \| .235*** \| .239*** \| .12 \| .113 \| \|  \| (.073) \| (.073) \| (.085) \| (.085) \| \| V. Impatient \| -.162 \| -.158 \| .038 \| .028 \| \|  \| (.184) \| (.186) \| (.215) \| (.215) \| \| Risk averse \| -.188* \| -.183 \| -.135 \| -.133 \| \|  \| (.114) \| (.115) \| (.123) \| (.123) \| \| Wrong answers \| -.145*** \| -.149*** \| -.126*** \| -.132*** \| \|  \| (.039) \| (.039) \| (.041) \| (.041) \| \| # Months \| .013 \| .015* \| .012 \| .012 \| \|  \| (.009) \| (.009) \| (.01) \| (.01) \| \| Germany \| .033 \| .029 \| .077 \| .075 \| \|  \| (.139) \| (.14) \| (.149) \| (.149) \| \| Poland \| .317** \| .3** \| .376** \| .381** \| \|  \| (.143) \| (.144) \| (.157) \| (.157) \| \| Observations \| 25293 \| 25293 \| 19592 \| 19592 \| \| Chi^2^ \| 147.142 \| 314.374 \| 43.69 \| 48.244 \| \| *Standard errors are in parentheses* \| \| \| \| \| \| **** p<.01, ** p<.05, * p<.1* \| \| \| \| \| \|  \| \| \| \| \| |
| --- | --- | --- | --- | --- | --- | --- | --- | --- | --- | --- | --- | --- | --- | --- | --- | --- | --- | --- | --- | --- | --- | --- | --- | --- | --- | --- | --- | --- | --- | --- | --- | --- | --- | --- | --- | --- | --- | --- | --- | --- | --- | --- | --- | --- | --- | --- | --- | --- | --- | --- | --- | --- | --- | --- | --- | --- | --- | --- | --- | --- | --- | --- | --- | --- | --- | --- | --- | --- | --- | --- | --- | --- | --- | --- | --- | --- | --- | --- | --- | --- | --- | --- | --- | --- | --- | --- | --- | --- | --- | --- | --- | --- | --- | --- | --- | --- | --- | --- | --- | --- | --- | --- | --- | --- | --- | --- | --- | --- | --- | --- | --- | --- | --- | --- | --- | --- | --- | --- | --- | --- | --- | --- | --- | --- | --- | --- | --- | --- | --- | --- | --- | --- | --- | --- | --- | --- | --- | --- | --- | --- | --- | --- | --- | --- | --- | --- | --- | --- | --- | --- | --- | --- | --- | --- | --- | --- | --- | --- | --- | --- | --- | --- | --- | --- | --- | --- | --- | --- | --- | --- | --- | --- | --- | --- | --- | --- | --- | --- | --- | --- | --- | --- | --- | --- | --- |

Multilevel (mixed *effects)* *two-way crossed-effects (subjects(i) X periods(j)) logit regression models on the binary dependent variable “Optimal Contract”, equal to one if the participant holds the best available contract in the current month, 0 otherwise. Columns 1 and 2 corresponds to the full sample (N=857, identical to* Table 4*) and columns 3 and 4 correspond to the sub-sample of active participants (those who switch at least once, N=636). Reminder is a dummy variable capturing the treatment effect; AfterAS1 is a dummy indicating the months after the first annual statement (i.e. month >12) ; ReminderXAS is the interaction of the two dummies. We control for Male,* *Fin. Literacy,* *Very impatient,* *Risk Aversion,* *Wrong Answers,* *,* country and total number of months as defined in Table 5. In Columns 2 and 4 we include the variable rem_AS_std capturing the triple interaction of Reminder, afterAS1 and Student and the variables rem_std and AS_std to capture the interactions of variables Reminder and afterAS1 with the variable student, respectively. *The panel dataset is unbalanced due to the fact that not all participants managed to solve all matrices and therefore go through all 40 months. Since we have a panel, the total number of observations is the product of the number of subjects (N) and the corresponding number of months for each subject. *** p<.01, ** p<.05, * p<.1*

Similarly, as shown in Figure C6b, analysis further revealed that active non-students do not make more optimal choice after receiving the reminder compared to autonomous switchers in the control. Indeed, switchers in the non-student reminder sample, fail to identify the second optimal switching point until they receive the annual statement in month 24, while autonomous non-student switchers in the control group identify the second optimal switching point more often. This effect compensates for the better performance of the non-students in the reminder treatment in the period between the first and second switching point.

Active students in the reminder treatment make significantly more optimal choices compared to active students in the control (*reminder+rem_std =0.245*, p =0.027, Table C6b column 4). The effect of the reminder in improving students’ choices is more evident after the second optimal switching point (yellow shadowed area).

1. Note that Circle was never optimal as Square provided the same conditions when participants had less than 325 points and better when they had more than 325 points, and selecting Circle to then switch to Square would have entailed some cost (i.e. time cost and 10 points switching fee). [↑](#footnote-ref-1)
